# Sex Hormones in Acquired Immunity and Autoimmune Disease

**DOI:** 10.3389/fimmu.2018.02279

**Published:** 2018-10-04

**Authors:** Vaishali R. Moulton

**Affiliations:** Division of Rheumatology, Department of Medicine, Beth Israel Deaconess Medical Center, Harvard Medical School, Boston, MA, United States

**Keywords:** hormones, estrogen, immune response, autoimmune disease, SLE

## Abstract

Women have stronger immune responses to infections and vaccination than men. Paradoxically, the stronger immune response comes at a steep price, which is the high incidence of autoimmune diseases in women. The reasons why women have stronger immunity and higher incidence of autoimmunity are not clear. Besides gender, sex hormones contribute to the development and activity of the immune system, accounting for differences in gender-related immune responses. Both innate and adaptive immune systems bear receptors for sex hormones and respond to hormonal cues. This review focuses on the role of sex hormones particularly estrogen, in the adaptive immune response, in health, and autoimmune disease with an emphasis on systemic lupus erythematosus.

## Introduction

From an evolutionary point of view, the paramount goal of all living organisms is to survive, reproduce and propagate the species. In humans and most vertebrates, the mother has the responsibility to bear the most vulnerable of the species—the offspring, and protect it from danger, to accomplish this supreme mission. Additionally there is non-genetic passive transfer of immunity from mother to offspring called trans-generational immune priming. Therefore, having the parental role may account for stronger immunity in females to defend and “prepare” for this responsibility. Intriguingly, the same immune response shifts during pregnancy to “tolerate” the foreign fetus and prevent rejection. Interestingly, in most fish species the father bears the parental responsibility. The Syngnathidae group includes seadragons, pipefish and the iconic seahorse. In these species, while it is the mother who produces the eggs, the father carries, nurtures the eggs through gestation, and gives birth to the young thus fulfilling the parental and immune priming role. There is evidence that there are differences in the immune response in the male seahorse during the parental vs. mating phases with improved immunity during the parental stage (1, 2). These observations suggest that the parental role comes with great immune power and responsibility. A “side-effect” of the stronger immune response is the higher propensity for developing autoimmune disease. This may be a plausible perspective to understand the gender bias of autoimmune disease.

Sex hormones not only control the reproductive system, but also regulate the development, and function of the immune response. Innate and adaptive, humoral and cell-mediated immune responses are impacted by hormones, and dysregulation of these mechanisms contribute to immune-mediated diseases including autoimmune disease (3–9). While the exact molecular mechanisms of how female hormones regulate the immune system are yet incompletely elucidated, studies show that they control development, homeostasis, gene expression, and signaling processes in T and B lymphocytes to influence their function in health and disease. This review focuses on the role of sex hormones on the adaptive immune system and in autoimmune diseases with a focus on the prototype systemic autoimmune disease SLE (10–12).

## Estrogen mechanisms of action

**Figure 1:**
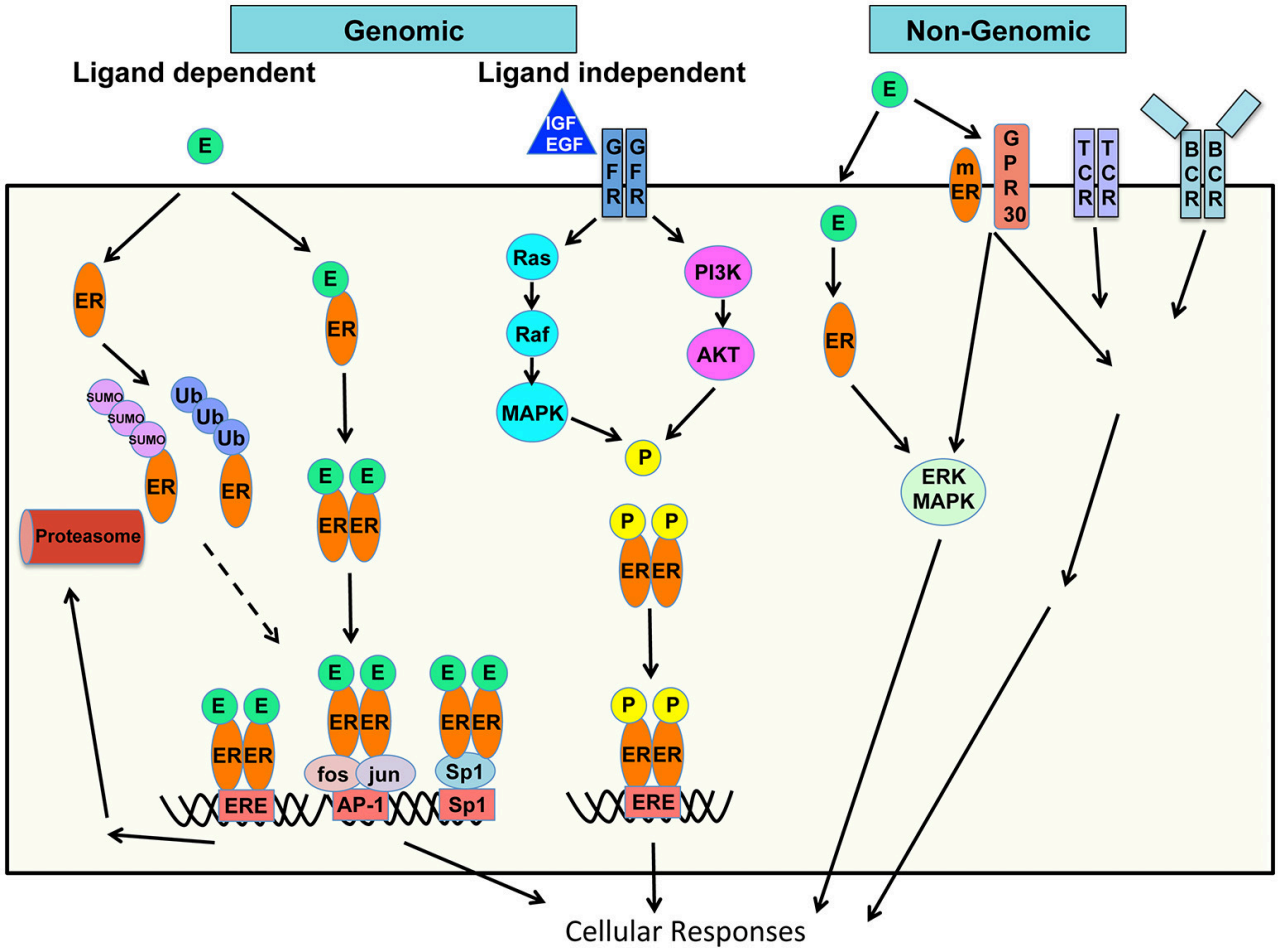
Schematic of mechanisms of estrogen action on cellular responses. Genomic and non-genomic, ligand dependent and ligand-independent, classic and non-classic receptor mediated estrogen-estrogen receptor signaling pathways are shown.

Estrogen acts via classical receptor-mediated, non-classical, and non-ligand-mediated genomic (nuclear) and non-genomic (extranuclear) pathways to control mechanisms of gene expression, protein modifications and signaling to influence cellular functions (Figure 1) (13–15).

### Genomic pathways of action

In the classical genomic pathway, Estrogen, or its most potent form 17-β-estradiol (E2) binds to its cognate intracellular steroid hormone receptor–estrogen receptor (ER). Two types of classical ER have been identified–ERα and ERβ encoded by the *Esr1* and *Esr2* genes respectively. The ER is a ligand-activated transcription factor, which bears ligand- and DNA-binding domains. Estrogen diffuses through the cell membrane, binds to cytoplasmic ER, which undergoes conformational change in the ER, and homo- or hetero-dimerizes. ER dimers then translocate into the nucleus and bind to promoters of target genes to regulate gene expression. In the non-classical genomic pathways, ER bound to DNA can interact with other transcription factors, or the ER may act in tether-mediated manner as co-factor with transcription factors including Specificity protein 1 (Sp1), activating protein 1 (AP-1), NF-κB and p300 proteins. ER/Sp1 and ER/AP-1 interactions activate a large number of genes and pathways and the ligand structure and specific ER-subtype dependent activation of either (16, 17). Activating functions (AF) 1 and 2 domains of the ERα bind to coregulators to regulate transcription and are both important in E2-mediated effects (18). When bound to the ligand, there is differential activation of the two ERs. Specifically ERα transactivates while ERβ inhibits transcription.

The ER binds specific motifs known as estrogen response elements (ERE) within the target DNA. The consensus ERE site is 5′-GGTCAnnnTGACC-3′ (19). While ERE sites within gene promoters are important in transcription, a chromatin Immunoprecipitation (ChIP)-paired end diTag cloning and sequencing whole genome cartography strategy identified ER binding sites in MCF-7 breast cancer cells and noted several interesting findings (20). Only 5% of mapped sites are in the proximal promoter regions of genes while a vast majority is in intronic or distal locations indicating transcriptional regulatory mechanisms over physical distances. Majority of the mapped sites were full ERE sites while 25% were half-sites and a small proportion (4%) had no recognizable ERE sequence (20). ERα and ERβ display dynamic interplay in their chromatin binding capacities and function. ERα and ERβ exhibit substantial overlap in the sites they can recognize, in cells that express either one of these receptors, whereas in cells that express both, fewer sites are shared. Cognate sites for both ERs are ERE-rich, however in cells that express both receptors ERα can competitively displace ERβ shifting it to new sites less enriched in ERE elements (21).

Besides being richly expressed in reproductive tissues, ERs are widely expressed in most cells in the immune system therefore influencing both innate and adaptive immune responses. There is age- and stage-dependent expression of ERs by lymphocyte precursors. Activated T cells express estrogen receptors (22) and both mRNA and protein levels of ER have been described for T cells, B cells, monocytes and dendritic cells. Differential expression of ER genes has been demonstrated in human peripheral blood mononuclear cells (PBMC) (23) and peripheral blood lymphocytes (PBL) (24). PBL CD4, CD8 T cells, B cells, and natural killer (NK) cells contain intracellular ER of which the ERα46 isoform is the most-expressed isoform. A cell surface ERα46 was detected in PBLs, and existence of a functional membrane (m) ERα was confirmed when a membrane-impermeant E2 mediated intracellular signaling activation and proliferation of T cells (24). CD4 T cells express high levels of ERα over ERβ while B cells express more ERβ than ERα mRNA. CD8 T cells and monocytes express low levels of both receptors (23).

ERα undergoes various posttranslational modifications including phosphorylation, acetylation, and ubiquitination, which modulate its stability and/or transcriptional activity. An interesting aspect of ER signaling and ER-mediated gene regulation is the continuous proteasome-mediated turnover of ERα. Estrogen can activate the Ubiquitin-Proteasome Pathway (UPP) to influence post-translational modifications and degradation of proteins. Ubiquitin is a small ~8 kDa protein which binds a series of three enzymes E1 (Ub-activating), E2 (Ub-carrier or conjugating), and E3 (Ub-ligase), which ultimately link it to the substrate protein. Ubiquitin-tagged proteins are targeted to the proteasome for degradation. This pathway is an important mechanism for tight control of the expression of short-lived inflammatory molecules and transcription factors including nuclear factor kappa B (NFκB), signal transducer and activator of transcription (STAT) 1 and cfos/jun to appropriately control their activity. Steroid hormone receptors including the ERs bind to protein components of the UPP including Ubc9, an E2 conjugating enzyme and E6-associated protein (E6-AP) which is an E3 ligase (25). Kruppel-like factor 5 (KLF5) is an important transcription factor, which inhibits cell proliferation, differentiation and carcinogenesis, and its levels are decreased in cancers including breast cancer. Estrogen induces the expression of estrogen responsive finger protein (EFP), an E3 ubiquitin ligase which leads to degradation of KLF5 in breast cancer cells (26). Similarly estrogen induces EFP-mediated degradation of another transcription factor tumor suppressor AT motif-binding factor 1 (ATBF1) which has an auto regulatory feedback with ERα signaling (27). Estrogen itself mediates downregulation of the ERα through the UPP (25, 28), and subsequently, the ERα mediated transcriptional activity and proteasomal degradation are inter-dependent. ERα was also shown to be a target for small ubiquitin-like modifier (SUMO)-1 modification (29). SUMOylation of the ERα hinge region is hormone-dependent and controls its transcriptional activity thus linking the estrogen and SUMO pathways. E3 ligases protein inhibitor of STAT1 (PIAS)1 and PIAS3 were shown to be E3 ligases for ERα (29), and addition of either Ubc9 or PIAS1 increased ERE-luciferase activity in COS cells (30).

Estrogen-independent functions of the ER include extensive phosphorylation, which control its transcriptional activity independently of its ligand. Environmental cues which activate the phosphoinositide 3-kinase/protein kinase B (PI3K)/Akt pathway and other kinases can phosphorylate the ER to regulate gene expression. ER independent functions of E2 were suggested in studies using ERα deficient wild-type (WT) or lupus-prone New Zealand Black × New Zealand White (NZB × NZW) founder 1 (F1) mice. A link between the ER and Toll-like receptor (TLR) signaling was shown as ERα deficiency led to reduced TLR9 signaling, reduced numbers of plasmacytoid dendritic cells (DC)s and impaired interferon (IFN)-α, interleukin (IL)-6, macrophage/monocyte chemoattractant protein (MCP)-1, IL-1β and IL-23 inflammatory cytokines (13).

### Non-genomic pathways of action

Besides the genomic pathway of gene regulation, estrogen can mediate effects through non-genomic mechanisms, through cross-talk with signaling cascades. Besides the classical intracellular ERs, Estrogen can bind to membrane estrogen receptors (mER) and membrane-associated G-protein coupled receptors (GPCRs) and trigger signaling downstream in certain cell types. Estrogen binds the G protein-coupled estrogen receptor 1 (GPER1) originally identified as G protein-coupled receptor 30 (GPR30) (31). These are also called rapid effects of estrogen mediated through membrane receptors, receptor tyrosine kinases, and signaling pathways downstream (31, 32). There is also transcriptional activation of genes by the GPER-induced response which include a first tier of transcription factors serum response factor (SRF), cyclic AMP repressor element binding protein (CREB), Ets family, then followed by a second tier including Fos, Jun, connective tissue growth factor (CTGF), early growth response protein (EGR)1, cyclic AMP dependent transcription factor (ATF)3, CCAAT/enhancer binding protein delta (C/EBPγ), and nuclear receptor related (NR)4A2 (33). Ligand induced activation of the mER and GPER can also integrate into intracellular signaling of the immune cell receptor such as the B cell receptor (BCR) signaling and activation pathways. Thus, non-nuclear non-genomic cytoplasmic effects of estrogen are attributed to increased calcium, through phospholipase C beta (PLCβ) activation, Gα and Gβγ protein activation, and kinase pathway activation including the mitogen activated protein kinase (MAPK), (PI3K) and mammalian target of rapamycin (mTOR) pathways (34, 35).

### Estrogen and microRNA in post-transcriptional gene regulation

In the last decade, the role of microRNA (miR) in post-transcriptional gene regulation has been uncovered as a powerful mechanism of gene regulation in health and disease as evidenced by the dramatic rise in the number of studies and publications in this field (36). miRs are short 22-nucleotide non-coding RNA molecules which are transcribed from genomic DNA and bind complementary sequences within the 3′untranslated region (UTR) of target genes to block translation or lead to degradation of the mRNA. miRs control genes involved in the immune response and aberrations in miR levels and activity can contribute to pathogenesis of autoimmune diseases. Therefore miRs are considered attractive biomarkers and targets for therapy. A large number (113) of miRs are encoded on the human X chromosome, second only to those on chromosome 1, which encodes 134 miRs, while the Y chromosome only encodes 2 miRs (37). Thus X-linked miRs likely contribute to the sex bias in autoimmunity. While the detailed functional characterization of all X-linked miRs in autoimmunity remains to be elucidated, a number of immune-suppressive genes are targeted by X-linked miRs including Forkhead box P3 (FoxP3), cytotoxic T lymphocyte associated protein 4 (CTLA4), Casitas B-lineage Lymphoma (CBL), CBL-B, suppressors of cytokine signaling (SOCS) genes, and programmed cell death 1 (PDCD1) as evidenced by putative predicted miR target sites within their 3′UTR (37). Besides the X-linked miR-mediated regulation, estrogen regulates microRNA expression to control genes of both innate and adaptive immune responses and therefore has implications for autoimmune disease (8, 36, 38, 39).

Estrogen upregulates miR-18a, miR-148a, miR-223, miR-451, miR-486, and miR-708, and downregulates SLE-linked miR-125, miR-145, and miR-146a. Microarray analysis showed that estrogen differentially regulates miRs in murine splenocytes *in vivo*. Treatment of mice with E decreases miR-146a and increases miR-223 which suppresses lipopolysaccharide (LPS)-induced IFN-γ and nitric oxide (NO) in splenic lymphocytes (40). miRs can also influence ER expression and modulate ER activity in disease (41). Estrogen activates STAT1-dependent transcriptional activation of TLR8 expression to promote inflammatory signaling via miR-21 in extracellular vesicles (42). A major role of estrogen is in bone remodeling and a protective role of estrogen is to suppress osteoclast mediated bone resorption. A novel mechanism by which estrogen preserves bone mass in bone marrow mesenchymal stem cells (BMMSC)s is to induce apoptosis of osteoclasts to protect from bone loss. Estrogen inhibits miR-181, which blocks FasL. Therefore estrogen promotes FasL protein expression by miR mediated posttranscriptional regulation in BMMSCs to maintain bone remodeling balance. In menopause, low estrogen levels, increased miR-181 and reduced FasL can promote survival of osteoclasts and increase bone loss (43).

## Estrogen and T lymphocytes

### T cell development

It is well known that estrogen suppresses T and B cell lymphopoiesis and activates B cell function. ERs are present on thymocytes as well as thymic epithelial cells (44). Estrogen influences T cell development and lymphopoiesis, and its effects on the thymus are complex. High doses of exogenous estrogen reduce thymic cellularity and cause thymic atrophy. This reduction is attributed to reduced proliferation of thymocytes precursors, both in the thymus and in the bone marrow (45). Accordingly, ovariectomy to remove the endogenous source of estrogen increases thymic cellularity with a shift to increased double positive (DP) thymocytes with reduced double negative (DN) and single positive (SP) cells (46). Conversely, estrogen treatment leads to reduced thymic cellularity with decreased proportions of DP cells (45, 47, 48), increased proportions of single positive (SP) CD4 and CD8 expressing variable beta chain (Vβ) T cell receptor (TCR), and alters distribution and TCRVβ expression of DN thymocytes (49). Pregnancy or treatment with estrogen induces a dramatic involution of the thymus (50–53). Estrogen mediates the loss of cortical thymocytes as evidenced by the reduced size of the thymic cortex in histological studies in mice (54, 55). Estrogen activates extrathymic T cell differentiation in the liver while inactivating intrathymic T cell development (48). However endogenous E2/ERα signaling is necessary for normal thymic size and function, because male and female ERα knockout (ko) mice still had reduced thymi and it was shown that ERα in non-hematopoietic tissues is essential for a normal full-sized thymus. Other receptor pathways are likely involved in estrogen-mediated thymic atrophy (56, 57), possibly due to increased E2 mediated effects through the ERβ or through effects on thymic stromal cells.

Besides thymocytes, sex-hormones also have varied effects on thymic epithelial cells (TEC) as evidenced by transcriptomics studies of cortical (c) and medullary (m) TECs in male, female, and castrated male mice. Male mice accumulated more cTECs but exhibited lower proliferation rates and expressed lower levels of genes involved in thymocyte expansion (58). The autoimmune regulator (Aire) gene is a transcriptional regulator important for expression of tissue specific antigens in mTECs for the positive and negative selection of T lymphocytes in the thymus. Thus Aire is a key molecule in central tolerance. In both mice and humans, reduced levels of Aire were found in females compared to males after puberty (59, 60). Estrogen downregulated Aire in cultured TECs, in human thyme grafted into mice, and in murine fetal thymic organ cultures by epigenetic modifications within the *Aire* promoter (60). Therefore estrogen-mediated regulation of T cell development and repertoire selection are important for central tolerance and contribute to autoimmunity.

### T cell homeostasis

The role of estrogen on cellular homeostasis is complex, depends on the cell/tissue type, concentrations of estrogen, and physiologic or pathological contexts (61). While physiologic concentrations of 17-β-estradiol stimulate survival and proliferation of cancer cells, and suppress apoptosis via Ras signaling in an ER dependent manner (62), pharmacological doses inhibit proliferation and induce apoptosis by ER independent pathways (63). Pharmacologic doses of 17-α-estradiol but not 17-β-estradiol induced G2/M cell cycle arrest in Jurkat cells which is exerted by ER independent mechanisms (64).

Estrogen stimulates growth and inhibits apoptosis a variety of cells but there is also evidence that estrogen induces apoptosis in breast cancer and other cells. Estrogen regulates apoptosis by both extrinsic Fas/FasL and intrinsic mitochondrial pathways (61). Culture of human PBMCs from healthy donors with 2-methoxyestradiol followed by pharmacologic phorbol myristic acid (PMA)/Ionomycin or physiologic CD3/CD28 stimulation led to decreased apoptosis and decreased Caspase-9 activity and reduced T cell proliferation with modest decreases in tumor necrosis factor (TNF) and IFN-γ production (65).

Ovariectomy in female albino oxford (AO) inbred rats led to an increase in the CD8 T cell compartment in peripheral blood and spleen, reflected in increased thymic double positive and CD8 cells and recent thymic emigrants (RTE) in peripheral blood. It also increased CD4+FoxP3+ CD4 T cells generation in the peripheral lymphoid tissues (66).

### T cell activation

Estrogen influences not only development but also various functions of T cells, in particular CD4 T cells including activation, cytokine production differentiation and regulatory functions with impact on physiology and autoimmune diseases (67, 68).

Estrogen and ERα are important in the activation, proliferation and pathogenic potential of T cells. T cell specific deletion of the ERα in mice led to transcriptomics changes with reduced expression of genes involved in T cell activation and reduced pathogenic potential in a T cell transfer model of colitis model (69). Estrogen downregulates DNA methyl transferase (DNMT) 1 expression and enhances global DNA hypomethylation in CD4 T cells from female SLE patients. While plasma β-estradiol levels were similar between patients and healthy controls, the mRNA expression of ERα but not ERβ was increased in SLE CD4 T cells (70). Aberrant extracellular regulated kinase (ERK)/MAPK signaling and resultant decrease in DNMT levels leading to DNA hypomethylation of a number of genes has been described and associated with autoimmune disease pathogenesis (71).

Estrogen controls immune cell activity through regulation of cellular metabolism via its receptors ERα, ERβ, membrane receptor mERα, mERβ, and GPER by direct and indirect mechanisms. The E2-ER-mediated control of transcription and signaling pathways stimulate mitochondrial function (72). The orphan nuclear receptor Estrogen related receptor (ERR) α controls transcription of a wide range of metabolic genes (73). ERRα was shown to control metabolic activity in T cells to influence T cell activation and is critical for Teffector (Teff) cell differentiation *in vivo* in ERRα-deficient mice. ERRα protein levels are low in resting T cells but increase upon activation. Glut1 upregulation, glucose uptake and mitochondrial processes were diminished in the absence of ERRα *in vivo* (74).

### T cell differentiation and cytokine production

Estrogen regulates a number of cytokines that modulate the immune response. Pharmacologic doses of the synthetic estrogen diethylstilbestrol in mice led to reduced proliferation of splenic T cells, reduced IL-2 production and increased susceptibility to *Listeria monocytogenes* infection (75). Estrogen increases NFκB signaling activity and its ensuing cytokines including IL-1, IL-10, and IFN-γ in C57Bl/6 mouse splenocytes (76). To assess the role of estrogen on T cell immune responses, concentration dependent effects of 17-β-estradiol *in vitro* cultures of T cells and splenocytes from rats were studied to assess the effects on proliferation, cytokines (IL-2 and IFN-γ), and signaling molecules ERK1/2, CREB, and Akt (77). Lower concentrations of estrogen enhanced proliferation and IFN-γ production in an ER dependent manner. The ERα agonist propyl pyrazole triol (PPT) suppressed IL-2, but the ERβ agonist diarylpropionitrile (DPN) increased IL-2. These effects were associated with increased levels of phosphorylated (p)-ERK, p-Akt and p-CREB and increased activity of antioxidant enzymes and NO production (77).

The luteal phase of the menstrual cycle in healthy young women associates with reduced IL-2 levels as evidenced by bioassay activity of serum IL-2 measurements as well as intracellular IL-2 within peripheral blood lymphocytes stimulated *ex vivo* (78). This decreased IL-2 may account for the observed increase in pre-menstrual infections or may presumably be a facet of the immune suppression necessary for a potential pregnancy. In human studies, E2 suppressed IL-2 production in T cells from healthy women and increased the expression of Sp1 transcription factor and the cAMP response element modulator (CREM) transcriptional repressor (79, 80). These studies showed that estrogen has specific concentration- and receptor-subtype dependent effects on immune responses.

Estrogen increases T helper cell (Th) 1 differentiation, IFN-γ and the inflammatory effects mediated by IFN-γ including production of inflammatory mediators inducible nitric oxide synthetase (iNOS), NO, and (cyclooxygenase) Cox2. Estrogen increases IFN-γ mRNA levels in murine splenocytes by activating the *IFN-*γ promoter, which contains consensus ERE sites as shown by promoter reporter assays in Jurkat cells (81). Administration of estrogen to ovariectomized Bagg Albino (BALB)/c mice followed by immunization with exogenous antigens increased antigen-specific clonal expansion of CD4 T cells and selectively increased Th1 cells and IFN-γ production. ERα on hematopoietic cells was necessary for the Th1 responsiveness (82). Further, estrogen was shown to upregulate the Th1 driving transcription factor T-box protein expressed in T cells (T-bet) in murine splenocytes by IL-27 and partly by IFN-γ but not by IL-12 (83). IL-12 signaling activates two isoforms of signal transducer and activator of transcription (STAT) 4, a full length STAT4α and a short STAT4β isoform. Estrogen selectively activates the short isoform (84).

Estrogen increased levels of IL-17 and its driving transcription factor retinoic acid receptor (ROR)γt in activated splenocytes from male and female C57Bl/6 wild-type mice and in lupus-prone male NZB/W mice. IL-27 and IFN-γ suppressed the IL-17 induction (85).

Other studies have shown the opposite effect of estrogen and ER on Th1 and Th17 cytokines and disease. In the experimental autoimmune encephalomyelitis (EAE) murine model of the CNS autoimmune disease multiple sclerosis (MS), estrogen mediates a neuroprotective effect (86), and suppresses Th1, Th17 responses. The estrogen-mediated inhibition of Th17 responses in this system is specifically via ERα expression on T cells (87). Estrogen suppresses IL-17 and Th17 differentiation in mouse CD4 T cells by downregulating the RORγt transcription factor mRNA and protein expression. This effect was mediated by an E2-activated complex of ERα and repressor of ER activity (REA) binding to three ERE half sites within the RORγt promoter (88). These studies indicate differential tissue-specific effects of estrogen on the immune response.

Estrogen is crucially important for its beneficial effects on bone metabolism, and postmenopausal estrogen decline is a critical factor in chronic inflammatory events including osteoporosis. IL-17 is implicated in the pathogenesis of inflammatory arthritis including RA and promotes bone loss in collagen-induced arthritis. In studies to assess the role of estrogen in IL-17 mediated regulation osteoclast and osteoblast differentiation, estrogen reversed the bone-destructing effects of IL-17. Therefore estrogen deficiency resulting in the de-repression of IL-17 may contribute to osteoporosis (89). Correspondingly, an evaluation of serum IL-17 levels in pre- and post-menopausal women showed a high prevalence of IL-17A levels in postmenopausal women, and inversely correlated with total lumbar T-scores, measures of bone loss (90). Estrogen also protects from bone loss through a transforming growth factor beta (TGF-β) signaling mediated pathway in T cells. TGF-β is an immunosuppressive cytokine and represses T cell activation, proliferation, and secretion of inflammatory cytokines. Accordingly, T cell specific TGF-β-signaling deficient mice had bone loss due to a de-repression of T cell activation and increased levels of osteoclastogenic cytokines TNF and receptor activator of NFκB ligand (RANKL) (91).

Peroxisome proliferator-activated receptor gamma (PPARγ) a nuclear receptor has recently been recognized as a critical regulator of adaptive immunity by negative regulation of T cell activation proliferation and differentiation. PPARγ mediated inhibition of Th1, Th2, and Th17 differentiation of naïve CD4 T cells from female C57Bl/6 mice whereas male cells only showed Th17 inhibition. Estradiol co-treatment of male cells inhibited Th1, Th2, and Th17 differentiation indicating that estrogen increases the sensitivity of male cells to the effects of PPARγ activation (92). Administration of the neurosteroid dehydroepiandrosterone (DHEA) inhibited Th17 responses and induced IL-10 producing regulatory cells in EAE and importantly reversed established paralysis and central nervous system (CNS) inflammation in mice. Further, DHEA-treated PBMCs from patients with relapsing remitting multiple sclerosis (RR-MS) exhibited decreased IFN-γ, IL-17, IL-4, and IL-2 responses but preserved IL-10. Thus such compounds, which suppress pro-inflammatory cells and expand regulatory subsets, could be useful as therapeutic agents (93).

### Regulatory T cells (Tregs)

Tregs are vitally important in the maintenance of self-tolerance and prevention of autoimmunity, and the X-linked master regulator transcription factor FoxP3 drives their generation, maintenance, and function (94, 95). Female gender and hormonal influences regulate FoxP3 expression and therefore are critical in the physiology of regulatory CD4 T cells and the gender bias of autoimmune disease (96). An imbalance between Teffs and Tregs is thought to contribute to dysregulated immune homeostasis and autoimmune disease.

In line with the observations that there is a maternal shift in the immune response to promote fetal tolerance, estrogen induced increased expression of CD25+ cells and increased FoxP3+ expression in naïve mice treated with Estrogen (97). Estrogen enhances Treg numbers and function, and induces FoxP3 expression both *in vitro* and *in vivo* (96). This effect is partially mediated through the checkpoint inhibitor programmed cell-death protein 1 (PD1). PD1 is a negative regulator of immune responses, is upregulated on activated T cells, considered a marker of dysfunctional T cells, is important for immune tolerance, and is an attractive target for autoimmune disease and cancer (98). Estrogen administration increased intracellular PD1 expression in CD4+FoxP3+ T cells, and PD1 expression was reduced in ER knockout mice (98).

Estrogen promoted the *ex vivo* proliferation of Tregs isolated from healthy human donors and also enhanced suppressive function in co-cultures with responder CD4+CD25- effector T cells (Teffs) (99). Increase in CD4+CD25+FoxP3+ T cells were observed in peripheral blood of fertile non-pregnant women in the late follicular phase of the menstrual cycle which correlated with β-estradiol levels, while there was a significant decline in Treg numbers in the luteal phase. Lower numbers of Tregs were found in follicular and luteal phases in women with recurrent spontaneous abortions (RSA) as well as from postmenopausal women. In addition Tregs from women with RSA also had reduced suppressive capacity compared to fertile women (100). Estrogen mediates its protective effect on bone metabolism through modulating Treg function on osteoclasts and bone resorption *in vitro* (101). E2 enhanced the suppressive capacity of Tregs on osteoclast differentiation from human embryonic bone marrow cells (BMC). Increased levels of both TGF-β1 and IL-10 suppressive cytokines were required for this effect because neutralizing both cytokines together but not individually, abolished the suppressive effect (101).

In Tregs derived from human cervical cancer tumor tissues, ERα blockade abolished FoxP3 expression and impaired suppressive function. ERE sites were found within the FoxP3 promoter ERα bound to the FoxP3 promoter in male blood-derived Tregs. Co-IP of E2 revealed E2-ERα complexes with FoxP3. Blocking with the anti-estrogen ICI 172 180 led to increase in IFN-γ & IL-4 production from Teffs derived from cervical-cancer suggesting that ER blockade could potentially restore certain Teff functions in tumors. These results showed that E2 and ERα are required for the FoxP3 expression and tumor-derived Treg and Teff function (102).

### T cell trafficking

Estrogen contributes to immune cell trafficking and inflammation by regulating chemokines and chemokine receptors. T cells from female mice displayed increased mRNA and protein expression of CC chemokine receptor (CCR) 1-CCR5 and increased transmigration response to chemokines macrophage inflammatory protein (MIP)-1β and stromal cell-derived factor (SDF)-1β. Similar increases in CCR gene expression were found in T cells from mice treated with estrogen *in vivo* (103). Estrogen increased the secretion of MCP-1, MCP-5, eotaxin, and SDF from mitogen activated splenocytes from estrogen treated mice (104). Further, *in vivo* trafficking of T cells was shown to be gender and estrogen-dependent.

Ovariectomized DBA/1 mice treated with estrogen and subjected to collagen-induced arthritis had fewer Th17 cells in the joints and less severe arthritis. However increased numbers of Th17 cells were found in the lymph nodes in early phase of disease, followed by a decrease in Th17 cells in the joints during established arthritis. Increased expression of CCR6 on the Th17 cells and corresponding increase in the chemokine CCL20 was though to contribute to interference with the egress of Th17 cells from lymph nodes to the joints indicating that estrogen modulates Th17 migratory pathways in inflammatory arthritis (105).

### T follicular helper (Tfh) cell function

Tfh cells provide cognate help to B cells to promote class switching and antibody production, and are implicated in autoantibody production in autoimmune diseases (106). Estrogen mediates gender-specific differences in regulation of Tfh cells responses via PPARγ. 4-hydroxy-3-nitrophenylacetyl hapten conjugated with ovalbumin (NP-Ova) immunization of female CD4/PPARγ deficient mice induced increased Tfh cells and germinal center (GC) B cells. Correspondingly treatment with a PPARγ agonist reduced responses in female and with E2 co-treatment in males (107). Estrogen increased Calcineurin and CD40 ligand (L) mRNA and protein expression in T cells from female SLE patients in an ER-dependent manner, therefore contributing to cognate B-cell help (108).

## Estrogen and B cells

Sex hormones play an important role in B cell development and function in physiology (109, 110) and contribute to their dysfunction in autoimmune disease (111). It has been known for a long time that estrogen enhances humoral responses, enhances B cell differentiation and immunoglobulin (Ig) production (112, 113).

### B cell development

Similar to its effects on thymic T cell development, estrogen suppresses B cell lymphopoiesis. Estrogen controls lymphoid-restricted progenitors in the bone marrow. Early B cell precursors are estrogen-sensitive and are decreased in the bone marrow during pregnancy and following estrogen administration in mice and humans. Specifically estrogen blocks B cell development at the differentiation step from pro-B cell to the pre B-cell stage (114–118). The E2-mediated inhibition of B lymphopoiesis is both due to a direct effect on B cells as well as on the stromal cells partially due to reduced production of the homeostatic cytokine IL-7 and increased expression of soluble frizzled related protein 1 (sFRP1) (119, 120).

### B cell homeostasis and activation

Besides lymphopoiesis and differentiation, estrogen regulates peripheral B cell populations, and tolerance induction by promoting survival and activation of autoreactive B cells (121, 122). In splenic populations estrogen treatment leads to increased marginal zone (MZ) B cells, reduced transitional B cells and slightly increased follicular B cells (111, 123–125). In BALB/c R4a mice transgenic for an anti-DNA antibody, E2 treatment led to increased serum anti-dsDNA antibodies, peripheral lymphoid expansion of high-affinity antibody-positive B cells, and increased expression of anti-apoptotic protein Bcl-2 in the germinal center B cells (126). Estrogen increased expression of activation genes including CD22 and SHP-1 and overexpression of these genes led to reduced B cell receptor (BCR) signaling (124). These DNA-reactive B cells escape deletion and E2 mediates rescue of autoreactive cells at the immature and transitional B cell stages. Specifically it was the high-affinity DNA-reactive B cells competitively survived in E2 treated mice compared with the low-affinity B cells in control mice (125). While both ERα and ERβ mediated B cell maturation, and CD22 expression, ERα was involved in the E-mediated decrease in BCR signaling, indicating differential roles of ERα and ERβ in B cell maturation vs. selection (127). Thus autoreactive B cell differentiation depends on the hormonal milieu wherein estrogen promotes marginal zone B cells (123), their long-term persistence and autoantibody secretion (128).

### B cell function

B lymphocyte stimulator (Blys) also called B cell activating factor (BAFF) is a vital cytokine for survival and maturation of B cells, and elevated serum levels have been found in SLE patients (129). Steady state mRNA and protein levels of BAFF were higher in immune cells from C57Bl/6 female mice and estrogen treatment increased BAFF expression which was mitigated in ERα, STAT1, or IRF5 deficient mice (130). Administration of β-estradiol by subcutaneous implants in NZB/W lupus-prone mice increased serum Blys levels, autoantibodies, and accelerated proteinuria and glomerulonephritis (131). In human studies, estrogen treatment led to increased BAFF mRNA levels in peripheral blood leukocytes from healthy men and women. Progesterone treatment increased BAFF mRNA in cells from women in a dose dependent manner, while lower concentrations increased but higher concentration decreased expression cells from men (132).

Besides the E2-mediated effect on B cell activation, which leads to increased immunoglobulin (Ig) antibody production from both bone marrow and splenic B cells, there is evidence of a direct effect of estrogen receptors on the Ig heavy chain locus. Specifically ERE were identified within the heavy chain switch (S) regions and an ERα antibody-mediated ChIP-sequencing (seq) analysis on genomic DNA from LPS-activated B cells revealed numerous ERα binding to key regulatory elements. These data support the idea that nuclear hormones and receptors can directly regulate class switch recombination and antibody expression (133).

In summary, estrogen mediates key effects on B cell physiology and function, which are vital in the pathogenesis of autoimmune diseases like SLE.

## Estrogen and autoimmune diseases

The female predilection of autoimmune diseases ranging from 3:1 for MS to 15:1 for autoimmune thyroiditis clearly implicates the female gender and sex hormones in autoimmunity (6, 8). While progesterone and androgens are considered immunosuppressive, therefore protective, estrogens in general are considered immune-stimulatory therefore pathogenic in autoimmune diseases. However, the role of estrogen is complicated and in some diseases, estrogens are immunostimulatory while in others they are inhibitory. There is an interesting dichotomy in the estrogen-mediated effects on different autoimmune diseases. While diseases like SLE worsen during pregnancy, others including MS, rheumatoid arthritis (RA), uveitis and thyroiditis improve, likely due to the maternal shift from a Th1 to Th2 immune response presumably as an attempt to avoid fetal rejection, and to enhance antibodies for passive transfer of immunity to the fetus. The diseases that are critically dependent on the T cell-dependent Th1 response, benefit from this diversion, while in SLE a shift to the Th2 propagates the autoantibody response to worsen disease.

## SLE

SLE is a prototypical chronic systemic autoimmune disease afflicting women in the childbearing years and can affect any organ in the body (10, 11). Joints and skin are frequently involved, while complications in vital organs such as kidneys can lead to lupus nephritis and renal failure. Complex interaction of genetics, environmental factors, and hormones lead to the deregulation and aberrant activation of the innate and adaptive immune systems leading to circulating autoantibodies and inflammatory immune cells which eventually lead to destruction of target organs (134, 135). Historically, studies with gonadectomy/hormone deprivation and hormone supplementation in male and female lupus prone mice have shown a clear association of sex hormones with lupus, where estrogen accelerates or worsens disease and estrogen removal ameliorates disease in females. Male gonad removal increases susceptibility to disease in male mice and androgen supplementation improves disease in female mice (6).

The role of ERs has been studied in various murine models of lupus. Ovariectomized NZB/W mice treated with the potent ERα agonist PPT developed increased levels of autoantibodies and proteinuria earlier and succumbed to disease sooner than control counterparts. However, the ERβ agonist DPN reduced some anti-dsDNA autoantibodies but not total IgG, proteinuria or mortality. These studies indicate that ERα has a pro-inflammatory role while ERβ has mild immunosuppressive effects in this system (136). Correspondingly, ERα deficiency attenuated autoantibodies and glomerulonephritis and improved survival in female and male (NZBxNZW) F1 mice (137). Another study found amelioration of disease in ERα-deficient female but not male NZM2410 and MRL/lpr strains of lupus-prone mice (138). Monthly injections of estradiol into ERα deficient mice induced a serum Th2 cytokine profile, increased kidney damage and death while minimal changes were observed in similar experiments conducted in ERα deficient mice (139).

Estrogen and ER signaling contribute to the activation or repression of a number of immunomodulatory cytokines, which contribute to disease pathogenesis and organ pathology in lupus (68). The murine lupus susceptibility locus *Sle1c2* is a sublocus of the NZM2410-derived Sle1 major lupus susceptibility locus and contributes to CD4 T cell activation, increased IFNγ-expressing T cells, and increased susceptibility to chronic graft vs. host disease (cGVHD), When crossed into the NZB lupus-prone mice, *Sle1c2* enhanced B cell activation, autoantibodies, and renal pathology. This locus contains the estrogen related receptor γ (*Esr*γ), expressed in T cells, which encodes for an orphan nuclear receptor that controls mitochondrial function and oxidative metabolism. B6Sle1c2 CD4 T cells expressed reduced levels of Esrγ, which correlated inversely with CD4 activation compared to B6 CD4 T cells. Increased levels of mediators of glycolysis, with reduced mitochondrial mass and membrane potential, but increased reactive oxygen intermediates (ROI) indicating mitochondrial dysfunction (140, 141). While global deficiency of ERα in lupus-prone B6.Sle1 mice ameliorates disease (142), conditional deletion utilizing the Cre-lox technology has shown the effect of ERα in specific immune cells. B cell specific deletion of ERα by crossing ERα flox mice with CD19-Cre mice delayed autoantibody production and lupus nephritis in (NZBxNZW) F1 lupus-prone mice (143).

SLE T cells display numerous defects in homeostasis, phenotype, signaling, metabolism, and function (12, 135, 144) and estrogen influences T cell signaling and activation in T cells from SLE patients. While serum estrogen levels *per se* have not been found to be significantly different in women with SLE, increased estrogen metabolism is observed. Higher levels of more feminizing estrone metabolites are observed in SLE patients and their first degree relatives implying that more potent metabolites may induce more potently epigenetic changes via the ERs (6, 145). ERα and ERα transcripts are expressed in PBMCs (146), and T cells from SLE patients and exhibit biologically active ER proteins binding to ERE sites (22). Differential expression of the ER subtypes and antibodies against ERs impact disease activity. Some studies have found alterations in ER expression with increased ERα mRNA levels but decreased ERαβ transcripts in PBMC from SLE patients (147). Others examined of intracellular ERα and ERβ in T cells showed much greater variability of expression of the ERs in SLE patients compared to healthy controls. ERα is implicated in a pro-inflammatory pathogenic role while ERβ has some anti-inflammatory roles in SLE. Polymorphisms in the ERα (*Esr*) gene have been linked with SLE and found to be significantly associated with the development of disease or age at disease onset, with a higher frequency in childhood-onset vs. adult onset patients or with disease features and severity (148–152).

ERK pathway downregulation and DNA hypomethylation are well-known underlying epigenetic aberrations in SLE (71, 153, 154). Estrogen suppressed ERK phosphorylation in *ex vivo* stimulated SLE T cells from patients with inactive or mild disease (155). In (C57Bl/6xSJL) F1 mice transgenic for a dominant negative MEK (dnMEK) selectively in T cells, estrogen led to ERK inactivation, DNA hypomethylation of the X-linked gene *CD40L*, and increased autoantibodies in female but not male mice. Estrogen-induces miR148a (39) which targets and suppresses DNMT1 expression in T cells leading to increased DNA hypomethylation (156). These results showed an effect of estrogen on epigenetic regulation of genes involved in disease pathophysiology (157).

The calcium-dependent phosphatase Calcineurin dephosphorylates nuclear factor of activated T cells (NFAT) to activate NFAT-mediated transcriptional activation of genes including the B-cell help molecule CD40L/CD154. Estrogen increases Calcineurin and CD154 expression levels in an ER dependent manner in T cells from women with SLE but not healthy controls (158, 159). Estradiol also increased the calcium-buffering protein Calreticulin in activated T cells from healthy donors but variably modulated it in activated T cells from SLE patients, suggesting a deregulated control in SLE T cells (160). Zinc finger acidic domain structure 3 (ZAS3) is a signaling and transcription factor, which regulates inflammatory responses. Increased ZAS3 mRNA and protein levels were found in PBMCs from SLE patients, and estradiol treatment increased ZAS3 expression levels in PBMCs and in mice injected with estradiol. ERα bound to ERE sites within the ZAS3 locus and was required for E2-mediated induction of ZAS3 (161).

Estrogen decreased activation induced cell death (AICD)-mediated apoptosis and downregulated FasL mRNA and protein expression in an ER-dependent manner in PMA-activated T cells *ex vivo* from SLE patients (162). Another study found that *in vitro* estradiol treatment of T cells from SLE patients led to increased expression of FasL and Caspase-8 but no change in Fas, Bcl-2, and Caspase-9 mRNA level (163). Thus the estrogen-mediated persistence of autoreactive cells may contribute to autoimmunity in SLE. Autoantibodies to ERα but not ERβ were identified in sera of about half of SLE patients tested, and ERα abs induced activation and apoptosis both in resting T cells and after CD3 activation. ERα autoantibody levels correlated with SLE disease activity index (SLEDAI) and arthritis clinical parameters (164) indicating that ERα autoantibodies disrupt T cell homeostasis in autoimmune disease.

Microarray gene profiles from activated T cells from female SLE patients and healthy controls showed alterations in a number of signaling pathways including Type I interferon, which has been clearly associated with disease initiation and progression. A Type I IFN gene altered was the vitamin D receptor interacting protein (DRIP150) suggesting that aberrant regulation of a cofactor may contribute to estradiol sensitivity in SLE T cells (165). Microarray analysis in PBMCs from SLE patients and healthy controls treated with estradiol revealed estrogen-mediated gene signatures. Many more genes were differentially regulated by estradiol in SLE T cells compared to healthy controls. Of note were pathways with genes involved in post-translational modification (161). A recent study utilized *in vitro* culture of T cells from female SLE patients or controls with the ER antagonist Fulvestrant/Faslodex (ICI 182, 780) to assess the global effects on estrogen-mediated genes signaling pathways by microarray gene profiling. Pathways of Th cell differentiation, steroid receptor (GR/ER) signaling, ubiquitination and sumoylation pathways were significantly altered. While the mRNA levels of both ERα and ERβ and protein levels of ERβ were similar, the protein expression of ERα in SLE T cells *ex vivo* was significantly lower in SLE compared to healthy controls suggesting an increased turnover (166). These studies suggest that increased turnover of ERα in SLE T cells may sensitize T cells to estradiol and contribute to their altered function.

In SLE, an imbalance between Th17 and Tregs is thought to contribute to and correlate with disease pathogenesis (167, 168). IL-6 is a crucial cytokine in this balance because IL-6 (with low dose TGFβ) drives naïve CD4 differentiation to Th17 cells, rather than Tregs (169), and inhibits TGFβ-induced Treg differentiation. High doses of TGFβ drive Treg differentiation. In addition, IL-6 in combination with IL-1β leads to degradation of FoxP3 (170). High serum and urine levels of IL-6 are found in SLE patients and correlate with disease activity (171–174). E2 stimulates IL-6 expression by biliary epithelial cells in mice and humans (175). IL-6 production is controlled genetically in an age- and gender dependent manner. In a human study (n.62, n.31 men and 31 women, aged 29 to 93 years), plasma IL-6 levels, IL-6 production by stimulated PBMC *ex vivo*, and a C to G transition at nucleotide−174 of the IL-6 gene promoter (−174 C/G locus) were assessed. Results showed that IL-6 production increases with age and is dominant in women (176). Accordingly, IL-6 knockout female C57BL/6 mice were resistant to syngeneic-activated lymphocyte-derived DNA (ALD-DNA)-induced SLE and IL-6 blockade increased FoxP3 expression, therefore showing that IL-6 suppresses Tregs to promote lupus (177). Thus IL-6 is a critical inflammatory cytokine, which shifts the balance from Tregs to Th17.

Type I as well as type II IFN cytokines are important in autoimmunity and inflammation (178, 179). Treatment of splenocytes from C57Bl/6 or lupus-prone NZB/W mice and murine cell lines with either IFN-α or IFN-γ led to increased expression of ERα mRNA and protein levels, via transcriptional activation of the *Esr1* promoter through STAT1. E2 and IFN signaling co-operatively activated ERα and IFN-responsive genes. These data bring to light a mutual positive regulatory feedback in which interferons activate ERα which activates IFN-γ and IFN-γ-mediated interferon regulatory factor (IRF) 9 to further amplify the inflammatory loop (180).

TNF-like weak inducer of apoptosis (TWEAK) is a TNF superfamily proinflammatory multifunctional cytokine, which can lead to increased inflammatory mediators including IL-6, MCP1 associated with renal damage in SLE (181). Higher urinary levels of soluble TWEAK were found in patients with renal damage compared to those without. Estrogen through ERα promotes expression of to accelerate the progression of lupus nephritis. E2 treatment of PBMCs from lupus nephritis (LN) patients led to increased mRNA levels of TWEAK, which were abolished in the presence of ERα inhibitor methyl-piperidino-pyrazole (MPP) and ER antagonist Fulvestrant (ICI 182 780). Similar results were obtained after ovariectomized MRL/lpr lupus-prone mice were treated with estrogen or antagonists. Severe renal pathology and high serum IL-6 levels in these mice were reversed by co-treatment *in vivo* with shRNA to inhibit TWEAK. (182). In C57BL/6 ERα knockout mice the nephrotoxic serum nephritis (NTN) model of immune-mediated nephropathy was used to assess the role of ERα in lupus nephritis. Time-course microarrays on murine glomeruli from wt and ERα-ko NTN-induced mice showed increased PPAR-γ mediated lipid metabolism and decreased retinol metabolic pathways. In parallel, RNA-seq analysis of whole blood from SLE patients revealed similar expression profiles of these pathways (183). Thus ERα signaling impacts metabolic activity in the kidneys to promote immune-mediated nephropathy and has implications for lupus nephritis.

These studies indicate that female hormones particularly estrogen plays important roles in immune cell generation, homeostasis, and function which impact control of immune responses. Caution must be exercised while interpreting data due the differences in systems studied, heterogeneity in patient populations, numbers and disease state of patients examined, and most importantly, concentrations and durations of estrogen exposure. Importantly, depletion of ERα and estrogen supplementation studies must be very carefully interpreted because most studies have been carried out with ERα knockout mice which have a functional rather than genetic ERα deficiency because they carry an N-terminal truncated form which lacks the critical AF-1 domain required for most classic estrogen actions. However ovariectomized true ERα–/– mice with genetic deletion of ERα in the NZM2410 strain, were not protected from lupus-like disease suggesting that other hormones perhaps testosterone mediate protection rather than the loss of full-length ERα (184).

## Other autoimmune diseases

While estrogen and ERs contribute to SLE pathogenesis and worsen disease activity in mice and humans, immune-protective effects are observed in other autoimmune diseases such as Multiple Sclerosis (MS) and rheumatoid arthritis (RA) (185).

### Multiple sclerosis

In MS, autoreactive T cells attack myelin tissue in the central nervous system leading to axonal demyelination and CNS dysfunction. Disease follows a relapse-remitting or progressive type of course. In this disease, both in humans and in the EAE mouse model, estrogen is neuroprotective by shifting the immune response and suppressing immune activation (186–189). Serial brain magnetic resonance imaging (MRI) during follicular and luteal phases of the menstrual cycles in eight women with relapsing-remitting MS showed significant correlation between Progesterone/β-estradiol ratios with both the numbers and volumes of lesions (190). A major clinical observation was that during pregnancy, the relapse rate of MS declines in the third trimester, but increases in the 3 months post-partum period (186). A pilot trial treatment of non-pregnant women with the pregnancy hormone estriol showed improvement in disease lesions (187). These effects are presumed to be due to the shift from a proinflammatory Th1 to anti-inflammatory Th2 immune response environment. Estrogen ameliorates EAE, and E2-ERα leads to reduced pro-inflammatory Th1, Th17 cells, and cytokines IFN-γ, IL-17, TNF, and other molecules iNOS and MCP-1. In addition Estrogen induces anti-inflammatory cytokines IL-10 and TGF-β and promotes expansion of Tregs. Estrogen suppresses CD4 T cell expansion, increases T cell apoptosis. E was shown to protect from atrophy of gray matter in EAE. ERα is shown to be pathogenic while ERβ is protective in MS. Accordingly ERβ ligand estriol administration was neuroprotective in EAE in mice (191). A new ERβ ligand AC186 improved reduced neuropathology in chronic EAE (192). A placebo-controlled multi-center Phase2b trial with oral ERβ ligand estriol improved disease activity (193), and another clinical trial is currently ongoing (www.clinicaltrials.gov).

E2 is protective in the EAE model of autoimmune disease in both male and ovariectomized female mice and this effect is partially mediated by modulation of Tregs (194). Estrogen upregulated PD1 expression in CD4+FoxP3+ Tregs, and PD1 levels rather than the frequency of Tregs, correlated with the degree of E2-mediated EAE protection. E2 also dramatically reduced IL-17 production, and this effect and protection from EAE were partially abrogated in the PD1ko mice (195). While PD1ko mice had normal FoxP3 expression levels, Tregs were functionally defective in their suppressive capacity which was partially restored by pre-treatment of the mice with Estrogen without much increase in FoxP3 levels. These results imply that estrogen influences Treg function via both PD1-dependent and independent pathways (196). EAE was suppressed in pregnant mice and in ovariectomized mice that received pregnancy levels of estrogen. Estrogen suppressed proliferation of T cells and decreased proinflammatory Th1 (IFN-γ, TNF-α) and Th17 (IL-17, IL-6) cytokine protein and mRNA levels while elevated Th2 (IL-4) and Treg suppressive (IL-10, TGF-β) cytokines in MOG-restimulated splenocytes and lymph node cells *ex vivo* from immunized mice. Accordingly, the respective transcription factors T-bet and RORγt were decreased while GATA3 binding protein (GATA3) and FoxP3 expression were increased (197).

### Rheumatoid arthritis

RA is the most common systemic rheumatic autoimmune disease and has a female to male incidence of 4:1 before the age of 50 and about 2:1 after the age of 60 years with the peak incidence around the fifth decade. Therefore female hormones clearly play a role in disease (198–200). However the contribution and effects of hormones in RA disease development are complicated and still not fully understood. Serum hormone levels fluctuate throughout the lifespan in women and interact differentially with genetic and environmental factors to regulate immune responses and autoimmunity. A number of factors are associated with the risk vs. protective effects of hormones in RA. Different hormonal states including pregnancy, post-partum, breastfeeding, and exogenous hormones including oral contraceptives (OC), postmenopausal hormone replacement therapy (HRT), and hormone administration for infertility treatment alter the hormonal milieu and are associated differentially with RA. Low estrogen levels such as earlier age at menopause, multi-parity, longer breastfeeding (>17 months) are associated with increased risk for RA. Pregnancy is protective for RA development and disease activity and so have HRT and OCs. Synovial tissues from RA patients have higher expression of the ERβ over ERα, and inflammation induces its expression to further induce proinflammatory cytokines TNF, IL-1β, and IL-6 by PBMCs (201). Diminished ovarian function and decreased circulating estrogen levels at menopause induces these cytokines and E2 inhibits them in PBMCs from postmenopausal women. However the E2-mediated effects on PBMC from pre-menopausal women are not consistent.

Therefore reduced estrogen bioavailability and decline in ovarian function contribute to development of RA. Hormones induce differential effects on immune system in pre-menopausal and post-menopausal women and therefore influence disease development differentially. The role of female hormones in the preclinical stages is still not fully understood (198).

## Hormones, receptor modulators, and related therapies

Because hormones play a critical role in physiology of reproductive tissue, bone, cardiovascular, lipid, and immune system, therefore contributing to disease pathogenesis, modulation of hormones or hormone receptors are considered therapies for cancer, bone diseases and autoimmune disease including SLE (202–204).

Effects of estrogen can be blocked by anti-estrogens or selective estrogen receptor modulators (SERM). Anti-estrogens include the pure ER antagonist Clomiphene citrate used for infertility treatment in anovulatory women and Fulvestrant (Faslodex) treatment of breast cancer. SERMs are synthetic estrogen-like ER-ligands, which have ER-agonistic or antagonistic effects depending on the target tissue without the adverse effects of steroid hormones. They have ER-agonistic effect on bone tissue, but minimal effects on reproductive tissues and are mainly used for their beneficial effects in postmenopausal vasomotor symptoms and osteoporosis. Tamoxifen is a first-generation SERM with competitive ER-antagonist effects on breast and agonist effects on bone, uterus and liver tissue. However, its uteroproliferative effects increase risk for endometrial cancer, negating its use for osteoporosis. Raloxifene, a second generation SERM is similar to Tamoxifen, but has anti-estrogen effects on breast and uterus but partial agonist in bone, lipids and cardiovascular system, and is approved for osteoporosis. Lasfoxifene and Bazedoxifene are third generation SERMs evaluated for their usefulness in osteoporosis (205).

A number of studies have assessed the effect of SERMs in bone loss in conjunction with effects on the immune system to assess their utility in postmenopausal osteoporosis. The effects of SERMs on the immune system are still being elucidated and some SERMs are shown to have immunoprotective effects. Continuous treatment with the selective estrogen receptor modulator (SERM) LY139478 ameliorated glomerulonephritis and improved survival in female MRL/lpr mice (206). MRL/lpr mice treated with the potent estrogen receptor antagonist Tamoxifen had reduced disease severity and decrease in numbers of double negative T cells and reduced IL-2 mediated DN cell proliferation *in vitro* (207). Oophorectomized normal mice treated with subcutaneous Raloxifene analog LY117018 had minimal changes on the thymus, T cell activity, and inflammation in DTH model indicating that Raloxifene does not exhibit similar effects as estrogen on T cell responsiveness and inflammation (208). Lasofoxifene and bazedoxifene are third generation SERMs with minimal estrogenic adverse effects used for treatment of postmenopausal osteoporosis. Similar to Raloxifene, Lasofoxifene, and Bazedoxifene did not increase peripheral B cell activity and only blocked B cell maturation at later stages of development therefore affecting fewer subpopulations, compared to estrogen treatment of ovariectomized female C57BL/6N mice indicating the safety of these drugs (209). A similar study assessed the effects of Raloxifene, Lasofoxifene, and Bazedoxifene on T cell development and T cell dependent inflammation (50). Raloxifene and Lasofoxifene but not Bazedoxifene reduced thymic weight but neither of these SERMS affected thymic T cell populations or delayed-type hypersensitivity (DTH) inflammation. Therefore Lasofoxifene and Bazedoxifene are safe to use because they do not impact T lymphopoiesis or T cell dependent inflammation (50).

Arctigenin is a plant-derived phytoestrogen SERM, considered a natural alternative to estrogen, and acts as a selective agonist of the immunosuppressive ERβ receptor. Arctigenin bound to and activated ERβ phosphorylation and nuclear translocation in the mouse EL4 T cell line, and inhibited mTORC1 activation and subsequent Th17 differentiation of naïve CD4 T cells from female C57BL/6 mice. This was associated with amelioration of dextran-sodium sulfate (DSS)-induced colitis in ovariectomized female C57BL/6 mice (210). A recent study showed that two novel SERMS (designated SERM2 and SERM7) and Raloxifene promoted anti-inflammatory signaling of CD14+ M2 type macrophages, diminished NFκB activity, induced the anti-inflammatory cytokine IL-10 and the IL-1R antagonist, and suppressed T cell proliferation (211).

Dehydroepiandrosterone (DHEA) is the natural steroid precursor of both androgens and estrogen in peripheral tissues. Increased metabolism of estrogen and reduced DHEA levels have been observed in SLE patients. Therefore treatment with DHEA is considered a therapeutic option for SLE (202). In a multicenter randomized double-blind placebo controlled clinical trial of adult women with SLE, Prasterone (generic DHEA) administration for 12 months was well-tolerated and improved or stabilized disease activity (212). Fulvestrant (Faslodex) a selective estrogen receptor downregulator and competitive inhibitor of estrogen was shown to improve SLE disease activity index (SLEDAI) scores and reduce T cell activation molecules CD154 and Calcineurin in a double-blind placebo-controlled trial in postmenopausal women with moderately active SLE (213).

Although female sex hormones are a culprit in the pathogenesis of autoimmune diseases such as SLE these hormones have vitally important beneficial effects on the reproductive system and bone metabolism. Therefore there are concerns about exogenous estrogen including the effects of hormone replacement therapy in post-menopausal women, oral contraceptives in pre-menopausal women, and hormone treatment for infertility, on disease activity in SLE (233, 234). A randomized, double-blind, placebo controlled trial evaluated the effect of combined estrogen-progesterone hormone replacement therapy in menopausal women inactive or stable-active SLE. Results from this trial showed increase in only mild to moderate but not severe flares compared to placebo and concluded that the benefits of HRT outweigh the small risk of flares in SLE (235). Similarly, combined oral contraceptives did not increase the risk of flares in women with stable disease activity in a double blind randomized noninferiority trial (236). A randomized placebo-controlled trial of another hormone replacement option Tibolone, a progestogen whose metabolites have affinity for the estrogen, progesterone and androgen receptors was conducted in postmenopausal women with inactive or controlled SLE. Tibolone was well tolerated and short-term use did not affect the frequency of flares (237). A pilot case-control prospective study investigated the immune-modulating effects of short-term controlled ovarian stimulation (COS) in infertile women to assess the effects of acute increase in E2 on serum BAFF levels, Immunoglobulins, anti-nuclear antibodies (ANA) and peripheral B cell phenotype and found no significant increases in these measures of immune activation suggesting the safety of COS in infertility treatment.

A modern HRT option is tissue-selective estrogen complex (TSEC) in which estrogen is combined with a SERM. In this therapy, the SERM competes for ER-binding in a tissue-specific manner to mediate protective effects on the tissue. An estrogen-Bazedoxifene combination was the first approved TSEC for prevention of postmenopausal vasomotor symptoms and osteoporosis and had better safety profiles and efficacy than conventional HRT, (238–241) and showed benefits by preventing bone loss in a collagen-induced arthritis (242). A study with E2 and Raloxifene showed suppressed E2-mediated autoreactive effects on B cells in NZB/NZW) F1 mice (243) However, the E2-Baze combination TSEC blocked uteroproliferation but did not affect the E2-mediated effects on thymus weight, or B lymphopoiesis or bone marrow B cell Ig secretion (244). Therefore, more studies of the role of TSECs in the immune system are needed to determine their usefulness.

ERβ is protective for bone loss and estrogen was shown to regulate bone marrow stromal cells senescence and stemness to prevent osteoporosis via ERβ and special AT-rich sequence binding protein 2 (SATB2) transcription factor. Estrogen induced ERβ-ERE binding to activate the promoter and upregulate SATB2. SATB2 ameliorated senescence, increased stemness and improved osteogenic differentiation of BMSCs from ovariectomized female SD rats (245). Therefore blocking estrogen or ERα are potential options, and targeting ERβ may be another potential avenue.

## Progesterone and androgens

While estrogen in general has immunostimulatory roles, Progesterone, and androgens are immunosuppressive and counteract the pathways affected by estrogen (214, 217). Progesterone receptors are present in lymphoid organs and cells of the innate and adaptive immune systems and are intracellular (iPR) or membrane bound (mPR) (215). Progesterone is shown to impact CD4 Th differentiation and cytokine production with increased IL-4, and increased Treg differentiation, and reduced IFN-γ, Th17 responses, reduced T cell proliferation and T cell-dependent antibody responses, in human peripheral blood and cell line or mouse studies. In CD8 T cells, Progesterone reduced IFN-γ and cytotoxicity. Effects on B cells included reduced class switch recombination and reduced T cell dependent antibody production (216).

Androgens also have immunosuppressive effects on the immune response (217). Low testosterone levels are correlated with higher B cells and antibody responses. Studies of gonadectomy or androgen receptor (AR) deficiency in male mice showed increased B lymphopoiesis, which was reversed by administration of testosterone. Overall, androgens promote B lymphopoiesis through B cell intrinsic mechanisms or effects on bone marrow stromal cells. Gonadectomized or AR deficient male mice have thymic atrophy, which returns to normal size after testosterone supplementation. Testosterone reduces the numbers of DP and CD4 SP cell and promotes CD8+ thymocytes presumably by inhibiting proliferation and increasing apoptosis. Testosterone increases the negative selection of autoreactive thymocytes by upregulating Aire expression in MTECs, and increases thymic TGFβ production therefore promoting central self-tolerance. Androgens also limit the peripheral lymphoid compartments and androgen deficiency or gonadectomy leads to increased peripheral lymphoid populations. Testosterone can non-selectively cause death of peripheral T cells. Effects of T cell responses are also observed in response to androgens. Removal of androgens leads to increased T cell responses, and treating female mice with testosterone reduces antigen-specific responses. Cytokine responses include a skewing toward the Th2 response with IL-4 and IL-10, and inhibiting Th1 differentiation, IL-12 and IFN-γ production. Testosterone promotes the expansion of Tregs and when ligand-bound, enhances FoxP3 expression in Tregs from rats or women in the ovulatory phase. Overall, androgens suppress the inflammatory responses of peripheral lymphoid cells through effects on T cells and indirect effects on B cells because peripheral B cells lack ARs (217). Although the incidence of SLE is far lower in men, disease is associated with poorer clinical outcomes in men. Indeed, testicular hypofunction was positively associated with SLE in a retrospective cohort study indicating that this requires consideration in patient management (218).

## Prolactin and leptin

Prolactin and Leptin influence the immune system and contribute to autoimmune diseases and inflammation. Prolactin is a luteotrophic hormone, which in general has immunostimulatory roles in the immune system. The reader is directed to an excellent review on Prolactin and autoimmunity within this topic collection (246).

Leptin, an adipocytokine is produced by adipose tissue and has dual roles as a hormone and a cytokine (219–223). As a hormone it impacts energy homeostasis, endocrine functions, and bone metabolism. As a cytokine, Leptin has multiple roles in the innate and adaptive immune responses, and promotes autoimmune and non-autoimmune inflammation. Leptin is in general, a proinflammatory molecule, which affects survival, activation, differentiation, and function of both T and B lymphocytes. Leptin promotes T cell survival and activation. It promotes IL-2 and IFN-γ production, and drives Th1 over Th2 differentiation (224). Leptin promotes expression of RORγt to drive Th17 differentiation in human and mouse CD4 T cells *in vitro* and *in vivo* (225). In contrast, Leptin suppresses Treg proliferation and expansion (230). Leptin is shown to activate the mTOR pathway and promote T cell glycolytic metabolism to regulate both Teffs proliferation and Tregs responsiveness (226, 227). In B cells, Leptin promotes expression of anti-apoptotic proteins Bcl-2 and Cyclin D1 to promote survival (231). Leptin activates JAK2/STAT3 and p38/MAPK/ERK1/2 signaling pathways in human B cells, and activates TNF, IL-6, IL-10 production (232).

Leptin is elevated in a number of autoimmune diseases including SLE (247), in humans and in murine models of lupus, and exerts pathogenic effects through increased Th17 proinflammatory responses, increased autoantibody production, impaired Treg responses, and increased availability of apoptotic cell-derived self-antigens (228, 229). Accordingly genetic deletion of leptin in mice, and the neutralization of leptin are shown to benefit autoimmune disease by restoring immune cell functions (228). Based on these findings, Leptin blockade may be considered a useful therapeutic approach for inflammatory diseases. However, downregulating effector immune responses would be detrimental during infections. Therefore, caution must be exercised in this direction, and appropriate selective targeting of molecules in the Leptin pathway may be considered better options.

Better understanding of the role of these hormones in immune responses and autoimmunity will pave the path for development for clinically relevant therapeutics to treat autoimmune diseases.

## Conclusions

The female gender-dependent bias in autoimmunity depends not only on the X chromosome but also the vast range of effects of sex hormones on the immune system and target organs. Sex hormones regulate molecular mechanisms in the innate and adaptive immune systems, and control immune responses in health. Complex interactions of hormones and environmental factors in genetically susceptible individuals lead to deregulation of the immune response, leading to immune-mediated diseases including autoimmune disease. While a large body of evidence exists for the role of estrogen in the immune response (Table 1), much remains to be learned. Complex roles of estrogen in different autoimmune diseases, with some protective roles in MS and RA, but pathogenic effects on others like SLE make it imperative to better understand the underlying basis for these dichotomies. Blocking estrogen receptors cautiously and in a targeted manner may yield better therapeutic outcomes than global treatment. Leptin is immunostimulatory, implicated in autoimmune disease, and targeting this hormone may be beneficial. Progesterone and androgens mediate immune-protective effects and therefore may be considered as potential therapeutic avenues.

**Table 1:** Effects of sex hormones on cells of the adaptive immune system.

**Hormone**	**Cells**	**Process**	**Effects**	**References**
Estrogen	T cells	Development	Suppresses thymopoiesis and thymic cellularity	(48–55)
			Activates extrathymic development in liver	(48)
			Downregulates Aire to impair negative selection of autoreactive T cells	(59, 60)
		Homeostasis (Physiologic conc)	Stimulates survival and proliferation and suppress apoptosis (cancer cells)	(61, 62)
		Homeostasis (Pharmacologic conc)	Reduces proliferation	(63–65)
		Activation	Increases T cell activation	(69)
			Increases NF-κB signaling	(76)
			Increases p-ERK, p-Akt, p-CREB signaling	(77)
			Stimulates mitochondrial function	(72, 74)
			Increases expression of Sp1 and CREM	(79)
			Impairs ERK/MAPK signaling, Decreases DNMT1, DNA hypomethylation	(71)
		Cytokine production	Reduces IL-2 (ERα), Increases IL-2 (ERβ)	(77–80)
			Increases IL-1, IL-10 IFN-γ	(81, 83, 84)
		Th Differentiation	Increases Th1 and Th17 differentiation Decreases Th2 differentiation	(82–85)
			Represses Th1, Th17, IFN-γ, IL-17 (Bone metabolism, CNS)	(87–90)
			Promotes TGF-β signaling (Bone metabolism)	(91)
		Tregs	Increases Treg numbers and FoxP3 expression	(96–99)
			Enhances Treg suppressive function	(99–102)
		T cell migration	Increases chemokine receptors CCR1-5	(103)
			Increases chemokines MCP1, MCP5, eotaxin and SDF1β	(104)
			Increases CCR6 on Th17 cells & chemokine CCL20; increases Th17 cell migration	(105)
		B cell help function (Tfh)	Increases Tfh cells	(107)
			Increases Calcineurin and CD40L expression	(108)
	B cells	Development	Suppresses B cell lymphopoiesis	(109, 110)
			Suppresses B cell differentiation from pro-B to pre-B cell stage	(114–120)
			Reduces threshold for negative selection; allows escape of autoreactive B cells	(126)
		Homeostasis/survival	Promotes survival of autoreactive B cells	(124, 125)
		Activation	Increases MZ and follicular B cells	(111, 123–125)
			Increases class switch and Ig antibody production	(128, 133)
		Cytokine production	Increases Blys (BAFF) levels	(129–131)
Progesterone	T cells	Homeostasis	Reduces T cell proliferation, Induces apoptosis	(214–216)
		Cytokine production	Increases IL-4, Decreases IFN-β, IL-17	
		Differentiation	Reduces Th1 Th17 differentiation	
		Function	Reduces T cell dependent antibody production	
			Inhibits cytotoxicity	
		Tregs	Increases Treg differentiation	
	B cells	Cytokine production	Promotes IL-10 production	
		Antibody production	Reduces class switch and T cell dependent antibody production	
Androgens	T cells	Development	Increase thymopoiesis	(217, 218)
			Increase Aire expression to promote deletion of autoreactive T cells	
		Differentiation	Inhibit Th1 and promotes Th2 and IL-10	
		Tregs	Increase FoxP3 and promotes Treg expansion	
	B cells	Development	Suppress B lymphopoiesis	
		Function	Reduce B cells and antibody responses	
Leptin	T cells	Activation and Differentiation	Promotes Th1 differentiation Increases RORγt, Promotes Th17	(219–225)
			Increases mTOR activation and proliferation of Teffs	(226, 227)
			Promotes Glycolysis to drive Teff differentiation	
			Increases availability of apoptotic cell-derived self-antigens, promotes autoimmunity	(228, 229)
		Tregs	Suppresses Treg proliferation and activity	(230)
	B cells	Homeostasis	Promotes survival by induction of Bcl-2 and Cyclin D1	(231)
		Activation	Increases JAK2/STAT3 and p38MAPK/ERK1/2	(232)
		Cytokine production	Increases TNF, IL-6, and IL-10	

## Author contributions

The author confirms being the sole contributor of this work and has approved it for publication.

### Conflict of interest statement

The author declares that the research was conducted in the absence of any commercial or financial relationships that could be construed as a potential conflict of interest.

## References

[1] Lin T Zhang D Liu X Xiao D. Parental care improves immunity in the seahorse (Hippocampus erectus). Fish Shellfish Immunol. (2016) 58:554–62. 10.1016/j.fsi.2016.09.065 27702678

[2] Keightley MC Wong BBM Lieschke GJ. Immune priming: mothering males modulate immunity. Curr Biol. (2013) 23:R76–8. 10.1016/j.cub.2012.11.050 23347945

[3] Klein SL Flanagan KL. Sex differences in immune responses. Nat Rev Immunol. (2016) 16:626–38. 10.1038/nri.2016.90 27546235

[4] Trombetta AC Meroni M Cutolo M. Steroids and autoimmunity. Front Horm Res. (2017) 48:121–32. 10.1159/000452911 28245457

[5] Edwards M Dai R Ahmed SA. Our environment shapes us: the importance of environment and sex differences in regulation of autoantibody production. Front Immunol. (2018) 9:478. 10.3389/fimmu.2018.00478 29662485 PMC5890161

[6] Lahita RG. The immunoendocrinology of systemic lupus erythematosus. Clin Immunol. (2016) 172:98–100. 10.1016/j.clim.2016.08.014 27546447

[7] Hughes GC Choubey D. Modulation of autoimmune rheumatic diseases by oestrogen and progesterone. Nat Rev Rheumatol. (2014) 10:740–51. 10.1038/nrrheum.2014.144 25155581

[8] Ortona E Pierdominici M Maselli A Veroni C Aloisi F Shoenfeld Y. Sex-based differences in autoimmune diseases. Ann Ist Super Sanita (2016) 52:205–12. 10.4415/ANN_16_02_12 27364395

[9] Kovats S. Estrogen receptors regulate innate immune cells and signaling pathways. Cell Immunol. (2015) 294:63–9. 10.1016/j.cellimm.2015.01.018 25682174 PMC4380804

[10] Tsokos GC. Systemic lupus erythematosus. N Engl J Med. (2011) 365:2110–21. 10.1056/NEJMra1100359 22129255

[11] Moulton VR Suarez-Fueyo A Meidan E Li H Mizui M Tsokos GC. Pathogenesis of human systemic lupus erythematosus: a cellular perspective. Trends Mol Med. (2017) 23:615–35. 10.1016/j.molmed.2017.05.006 28623084 PMC5650102

[12] Katsuyama T Tsokos GC Moulton VR. Aberrant T cell signaling and subsets in systemic lupus erythematosus. Front Immunol. (2018) 9:1088. 10.3389/fimmu.2018.01088 29868033 PMC5967272

[13] Cunningham M Gilkeson G. Estrogen receptors in immunity and autoimmunity. Clin Rev Allergy Immunol. (2011) 40:66–73. 10.1007/s12016-010-8203-5 20352526

[14] Nilsson S Mäkelä S Treuter E Tujague M Thomsen J Andersson G. Mechanisms of estrogen action. Physiol Rev. (2001) 81:1535–65. 10.1152/physrev.2001.81.4.1535 11581496

[15] Hall JM Couse JF Korach KS. The multifaceted mechanisms of estradiol and estrogen receptor signaling. J Biol Chem. (2001) 276:36869–72. 10.1074/jbc.R100029200 11459850

[16] Paech K Webb P Kuiper GG Nilsson S Gustafsson J Kushner PJ. Differential ligand activation of estrogen receptors ERα and ERβ at AP1 sites. Science (1997) 277:1508–10. 9278514 10.1126/science.277.5331.1508

[17] Safe S Kim K Kim K. Non-classical genomic estrogen receptor (ER)/specificity protein and ER/activating protein-1 signaling pathways. J Mol Endocrinol. (2008) 41:263–75. 10.1677/JME-08-0103 18772268 PMC2582054

[18] Andersson A Törnqvist AE Moverare-Skrtic S Bernardi AI Farman HH Chambon P. Roles of activating functions 1 and 2 of estrogen receptor α in lymphopoiesis. J Endocrinol. (2018) 236:99–109. 10.1530/JOE-17-0372 29255084

[19] Klinge CM. Estrogen receptor interaction with estrogen response elements. Nucleic Acids Res. (2001) 29:2905–19. 11452016 10.1093/nar/29.14.2905 PMC55815

[20] Lin C-Y Vega VB Thomsen JS Zhang T Kong SL Xie M. Whole-genome cartography of estrogen receptor α binding sites. PLoS Genet. (2007) 3:e87. 10.1371/journal.pgen.0030087 17542648 PMC1885282

[21] Charn TH Liu ET-B Chang EC Lee YK Katzenellenbogen JA Katzenellenbogen BS. Genome-wide dynamics of chromatin binding of estrogen receptors α and β: mutual restriction and competitive site selection. Mol Endocrinol. (2010) 24:47–59. 10.1210/me.2009-0252 19897598 PMC2802902

[22] Suenaga R Rider V Evans MJ Abdou NI. *In vitro-*activated human lupus T cells express normal estrogen receptor proteins which bind to the estrogen response element. Lupus (2001) 10:116–22. 10.1191/096120301673870511 11237123

[23] Phiel KL Henderson RA Adelman SJ Elloso MM. Differential estrogen receptor gene expression in human peripheral blood mononuclear cell populations. Immunol Lett. (2005) 97:107–13. 10.1016/j.imlet.2004.10.007 15626482

[24] Pierdominici M Maselli A Colasanti T Giammarioli AM Delunardo F Vacirca D. Estrogen receptor profiles in human peripheral blood lymphocytes. Immunol Lett. (2010) 132:79–85. 10.1016/j.imlet.2010.06.003 20542061

[25] Lonard DM Nawaz Z Smith CL O'Malley BW. The 26S proteasome is required for estrogen receptor-α and coactivator turnover and for efficient estrogen receptor-α transactivation. Mol Cell (2000) 5:939–48. 10.1016/S1097-2765(00)80259-2 10911988

[26] Zhao K-W Sikriwal D Dong X Guo P Sun X Dong J-T. Oestrogen causes degradation of KLF5 by inducing the E3 ubiquitin ligase EFP in ER-positive breast cancer cells. Biochem J. (2011) 437:323–33. 10.1042/BJ20101388 21542805 PMC3733548

[27] Dong X-Y Fu X Fan S Guo P Su D Dong J-T. Oestrogen causes ATBF1 protein degradation through the oestrogen-responsive E3 ubiquitin ligase EFP. Biochem J. (2012) 444:581–90. 10.1042/BJ20111890 22452784 PMC3754848

[28] Reid G Hübner MR Métivier R Brand H Denger S Manu D. Cyclic, proteasome-mediated turnover of unliganded and liganded ERα on responsive promoters is an integral feature of estrogen signaling. Mol Cell. (2003) 11:695–707. 10.1016/S1097-2765(03)00090-X 12667452

[29] Sentis S Le Romancer M Bianchin C Rostan M-C Corbo L. Sumoylation of the estrogen receptor α hinge region regulates its transcriptional activity. Mol Endocrinol. (2005) 19:2671–84. 10.1210/me.2005-0042 15961505

[30] Kobayashi S Shibata H Yokota K Suda N Murai A Kurihara I. FHL2, UBC9, and PIAS1 are novel estrogen receptor α-interacting proteins. Endocr Res. (2004) 30:617–21. 10.1081/ERC-200043789 15666801

[31] Revankar CM Cimino DF Sklar LA Arterburn JB Prossnitz ER. A transmembrane intracellular estrogen receptor mediates rapid cell signaling. Science (2005) 307:1625–30. 10.1126/science.1106943 15705806

[32] Barton M Filardo EJ Lolait SJ Thomas P Maggiolini M Prossnitz ER. Twenty years of the G protein-coupled estrogen receptor GPER: historical and personal perspectives. J Steroid Biochem Mol Biol. (2018) 176:4–15. 10.1016/j.jsbmb.2017.03.021 28347854 PMC5716468

[33] Maggiolini M Picard D. The unfolding stories of GPR30, a new membrane-bound estrogen receptor. J Endocrinol. (2010) 204:105–14. 10.1677/JOE-09-0242 19767412

[34] Seto K Hoang M Santos T Bandyopadhyay M Kindy MS Dasgupta S. Non-genomic oestrogen receptor signal in B lymphocytes: an approach towards therapeutic interventions for infection, autoimmunity and cancer. Int J Biochem Cell Biol. (2016) 76:115–8. 10.1016/j.biocel.2016.04.018 27189345

[35] Stefkovich ML Arao Y Hamilton KJ Korach KS. Experimental models for evaluating non-genomic estrogen signaling. Steroids (2018) 133:34–7. 10.1016/j.steroids.2017.11.001 29122548 PMC5864539

[36] Klinge CM. Estrogen action: receptors, transcripts, cell signaling, and non-coding RNAs in normal physiology and disease. Mol Cell Endocrinol. (2015) 418(Pt 3):191–2. 10.1016/j.mce.2015.11.028 26681526

[37] Hewagama A Role of X-Chromosome encoded miRNAs in Autoimmunity: suppressing the suppressor and female predisposition. Rheumatol Curr Res. (2013) 03:118 10.4172/2161-1149.1000118

[38] Dai R Ahmed SA. Sexual dimorphism of miRNA expression: a new perspective in understanding the sex bias of autoimmune diseases. Ther Clin Risk Manag. (2014) 10:151–63. 10.2147/TCRM.S33517 24623979 PMC3949753

[39] Honarpisheh M Köhler P von Rauchhaupt E Lech M. The involvement of MicroRNAs in modulation of innate and adaptive immunity in systemic lupus erythematosus and lupus nephritis. J Immunol Res. (2018) 2018:4126106. 10.1155/2018/4126106 29854836 PMC5964414

[40] Dai R Phillips RA Zhang Y Khan D Crasta O Ahmed SA. Suppression of LPS-induced Interferon-gamma and nitric oxide in splenic lymphocytes by select estrogen-regulated microRNAs: a novel mechanism of immune modulation. Blood (2008) 112:4591–7. 10.1182/blood-2008-04-152488 18791161 PMC2597130

[41] Ma X Liu Q. MicroRNAs in the pathogenesis of systemic lupus erythematosus. Int J Rheum Dis. (2013) 16:115–21. 10.1111/1756-185X.12083 23773633

[42] Young NA Valiente GR Hampton JM Wu L-C Burd CJ Willis WL. Estrogen-regulated STAT1 activation promotes TLR8 expression to facilitate signaling via microRNA-21 in systemic lupus erythematosus. Clin Immunol. (2017) 176:12–22. 10.1016/j.clim.2016.12.005 28039018 PMC5815376

[43] Shao B Liao L Yu Y Shuai Y Su X Jing H. Estrogen preserves Fas ligand levels by inhibiting microRNA-181a in bone marrow-derived mesenchymal stem cells to maintain bone remodeling balance. FASEB J. (2015) 29:3935–44. 10.1096/fj.15-272823 26062603

[44] Savino W Mendes-da-Cruz DA Lepletier A Dardenne M. Hormonal control of T-cell development in health and disease. Nat Rev Endocrinol. (2016) 12:77–89. 10.1038/nrendo.2015.168 26437623

[45] Zoller AL Kersh GJ. Estrogen induces thymic atrophy by eliminating early thymic progenitors and inhibiting proliferation of β-selected thymocytes. J Immunol. (2006) 176:7371–8. 10.4049/jimmunol.176.12.7371 16751381

[46] Leposavić G Karapetrović B Obradović S Vidiíc Dandović B Kosec D. Differential effects of gonadectomy on the thymocyte phenotypic profile in male and female rats. Pharmacol Biochem Behav. (1996) 54:269–76. 8728568 10.1016/0091-3057(95)02165-5

[47] Okasha SA Ryu S Do Y McKallip RJ Nagarkatti M Nagarkatti PS. Evidence for estradiol-induced apoptosis and dysregulated T cell maturation in the thymus. Toxicology (2001) 163:49–62. 10.1016/S0300-483X(01)00374-2 11376864

[48] Okuyama R Abo T Seki S Ohteki T Sugiura K Kusumi A. Estrogen administration activates extrathymic T cell differentiation in the liver. J Exp Med. (1992) 175:661–9. 1531494 10.1084/jem.175.3.661 PMC2119148

[49] Screpanti I Meco D Morrone S Gulino A Mathieson BJ Frati L *In vivo* modulation of the distribution of thymocyte subsets: effects of estrogen on the expression of different T cell receptor Vβ gene families in CD4-, CD8- thymocytes. Cell Immunol. (1991) 134:414–26. 1708703 10.1016/0008-8749(91)90314-2

[50] Bernardi AI Andersson A Stubelius A Grahnemo L Carlsten H Islander U. Selective estrogen receptor modulators in T cell development and T cell dependent inflammation. Immunobiology (2015) 220:1122–8. 10.1016/j.imbio.2015.05.009 26044996

[51] Clarke AG Kendall MD. The thymus in pregnancy: the interplay of neural, endocrine and immune influences. Immunol Today (1994) 15:545–51. 10.1016/0167-5699(94)90212-7 7802926

[52] Hirahara H Ogawa M Kimura M Iiai T Tsuchida M Hanawa H. Glucocorticoid independence of acute thymic involution induced by lymphotoxin and estrogen. Cell Immunol. (1994) 153:401–11. 10.1006/cimm.1994.1038 8118872

[53] Marotti T Sirotković M Pavelić J Gabrilovac J Pavelić K. *In vivo* effect of progesteron and estrogen on thymus mass and T-cell functions in female mice. Horm Metab Res. (1984) 16:201–3. 10.1055/s-2007-1014742 6609869

[54] Kendall MD Clarke AG. The thymus in the mouse changes its activity during pregnancy: a study of the microenvironment. J Anat. (2000) 197(Pt 3):393–411. 10.1046/j.1469-7580.2000.19730393.x 11117626 PMC1468141

[55] Erlandsson MC Ohlsson C Gustafsson JA Carlsten H. Role of oestrogen receptors α and β in immune organ development and in oestrogen-mediated effects on thymus. Immunology (2001) 103:17–25. 10.1046/j.1365-2567.2001.01212.x 11380688 PMC1783216

[56] Staples JE Gasiewicz TA Fiore NC Lubahn DB Korach KS Silverstone AE. Estrogen receptor α is necessary in thymic development and estradiol-induced thymic alterations. J Immunol. (1999) 163:4168–74. 10510352

[57] Yellayi S Teuscher C Woods JA Welsh TH Tung KS Nakai M. Normal development of thymus in male and female mice requires estrogen/estrogen receptor-α signaling pathway. Endocrine (2000) 12:207–13. 10.1385/ENDO:12:3:207 10963039

[58] Dumont-Lagacé M St-Pierre C Perreault C. Sex hormones have pervasive effects on thymic epithelial cells. Sci Rep. (2015) 5:12895. 10.1038/srep12895 26250469 PMC4528223

[59] Bakhru P Su MA Estrogen turns down “the AIRE.” J Clin Invest. (2016) 126:1239–41. 10.1172/JCI86800 26999606 PMC4811114

[60] Dragin N Bismuth J Cizeron-Clairac G Biferi MG Berthault C Serraf A. Estrogen-mediated downregulation of AIRE influences sexual dimorphism in autoimmune diseases. J Clin Invest. (2016) 126:1525–37. 10.1172/JCI81894 26999605 PMC4811157

[61] Lewis-Wambi JS Jordan VC. Estrogen regulation of apoptosis: how can one hormone stimulate and inhibit? Breast Cancer Res. (2009) 11:206. 10.1186/bcr2255 19519952 PMC2716493

[62] Fernando RI Wimalasena J. Estradiol abrogates apoptosis in breast cancer cells through inactivation of BAD: Ras-dependent nongenomic pathways requiring signaling through ERK and Akt. Mol Biol Cell. (2004) 15:3266–84. 10.1091/mbc.e03-11-0823 15121878 PMC452582

[63] Jenkins JK Suwannaroj S Elbourne KB Ndebele K McMurray RW. 17-β-estradiol alters Jurkat lymphocyte cell cycling and induces apoptosis through suppression of Bcl-2 and cyclin A. Int Immunopharmacol. (2001) 1:1897–911. 10.1016/S1567-5769(01)00114-X 11606022

[64] Jun DY Park HS Kim JS Kim JS Park W Song BH. 17α-estradiol arrests cell cycle progression at G2/M and induces apoptotic cell death in human acute leukemia Jurkat T cells. Toxicol Appl Pharmacol. (2008) 231:401–12. 10.1016/j.taap.2008.05.023 18603276 PMC2853923

[65] Luc JGY Jackson K Weinkauf JG Freed DH Nagendran J. Feasibility of lung transplantation from donation after circulatory death donors following portable *ex vivo* lung perfusion: a pilot study. Transplant Proc. (2017) 49:1885–92. 10.1016/j.transproceed.2017.04.010 28923643

[66] Arsenović-Ranin N Kosec D Nacka-Aleksić M Pilipović I Stojić-Vukanić Z Djikić J. Ovarian hormone level alterations during rat post-reproductive life-span influence CD8^+^ T-cell homeostasis. Exp Biol Med. (2015) 240:1319–32. 10.1177/1535370215570817 25716018 PMC4935252

[67] Pernis AB. Estrogen and CD4^+^ T cells. Curr Opin Rheumatol. (2007) 19:414–20. 10.1097/BOR.0b013e328277ef2a 17762604

[68] Kassi E Moutsatsou P. Estrogen receptor signaling and its relationship to cytokines in systemic lupus erythematosus. J Biomed Biotechnol. (2010) 2010:317452. 10.1155/2010/317452 20617147 PMC2896666

[69] Mohammad I Starskaia I Nagy T Guo J Yatkin E Väänänen K. Estrogen receptor α contributes to T cell-mediated autoimmune inflammation by promoting T cell activation and proliferation. Sci Signal. (2018) 11:9415. 10.1126/scisignal.aap9415 29666308

[70] Wu Z Sun Y Mei X Zhang C Pan W Shi W. 17β-oestradiol enhances global DNA hypomethylation in CD4-positive T cells from female patients with lupus, through overexpression of oestrogen receptor-α-mediated downregulation of DNMT1. Clin Exp Dermatol. (2014) 39:525–32. 10.1111/ced.12346 24825143

[71] Richardson B. The interaction between environmental triggers and epigenetics in autoimmunity. Clin Immunol. (2018) 192:1–5. 10.1016/j.clim.2018.04.005 29649575 PMC5988979

[72] Klinge CM. Estrogens regulate life and death in mitochondria. J Bioenerg Biomembr. (2017) 49:307–24. 10.1007/s10863-017-9704-1 28401437

[73] Giguère V. Transcriptional control of energy homeostasis by the estrogen-related receptors. Endocr Rev. (2008) 29:677–96. 10.1210/er.2008-0017 18664618

[74] Michalek RD Gerriets VA Nichols AG Inoue M Kazmin D Chang C-Y. Estrogen-related receptor-α is a metabolic regulator of effector T-cell activation and differentiation. Proc Natl Acad Sci USA. (2011) 108:18348–53. 10.1073/pnas.1108856108 22042850 PMC3215012

[75] Pung OJ Tucker AN Vore SJ Luster MI. Influence of estrogen on host resistance: increased susceptibility of mice to Listeria monocytogenes correlates with depressed production of interleukin 2. Infect Immun. (1985) 50:91–6. 3876288 10.1128/iai.50.1.91-96.1985 PMC262140

[76] Dai R Phillips RA Ahmed SA. Despite inhibition of nuclear localization of NF-κ B p65, c-Rel, and RelB, 17-β estradiol up-regulates NF-κ B signaling in mouse splenocytes: the potential role of Bcl-3. J Immunol. (2007) 179:1776–83. 10.4049/jimmunol.179.3.1776 17641044

[77] Priyanka HP Krishnan HC Singh RV Hima L Thyagarajan S. Estrogen modulates *in vitro* T cell responses in a concentration- and receptor-dependent manner: effects on intracellular molecular targets and antioxidant enzymes. Mol Immunol. (2013) 56:328–39. 10.1016/j.molimm.2013.05.226 23911387

[78] Trzonkowski P Myśliwska J Tukaszuk K Szmit E Bryl E Myśliwski A. Luteal phase of the menstrual cycle in young healthy women is associated with decline in interleukin 2 levels. Horm Metab Res. (2001) 33:348–53. 10.1055/s-2001-15420 11456283

[79] Moulton VR Holcomb DR Zajdel MC Tsokos GC. Estrogen upregulates cyclic AMP response element modulator α expression and downregulates interleukin-2 production by human T lymphocytes. Mol Med. (2012) 18:370–8. 10.2119/molmed.2011.00506 22281835 PMC3356426

[80] Moulton VR Tsokos GC. Why do women get lupus? Clin Immunol. (2012) 144:53–6. 10.1016/j.clim.2012.04.003 22659035

[81] Fox HS Bond BL Parslow TG. Estrogen regulates the IFN-gamma promoter. J Immunol. (1991) 146:4362–7. 1904081

[82] Maret A Coudert JD Garidou L Foucras G Gourdy P Krust A. Estradiol enhances primary antigen-specific CD4 T cell responses and Th1 development *in vivo*. essential role of estrogen receptor α expression in hematopoietic cells. Eur J Immunol. (2003) 33:512–21. 10.1002/immu.200310027 12645950

[83] Karpuzoglu E Phillips RA Gogal RM Ansar Ahmed S IFN-gamma-inducing transcription factor, T-bet is upregulated by estrogen in murine splenocytes: role of IL-27 but not IL-12. Mol Immunol. (2007) 44:1808–14. 10.1016/j.molimm.2006.08.005 17046061 PMC3097111

[84] Karpuzoglu E Phillips RA Dai R Graniello C Gogal RM Ahmed SA. Signal transducer and activation of transcription (STAT) 4β, a shorter isoform of interleukin-12-induced STAT4, is preferentially activated by estrogen. Endocrinology (2009) 150:1310–20. 10.1210/en.2008-0832 18988675 PMC2654738

[85] Khan D Dai R Karpuzoglu E Ahmed SA. Estrogen increases, whereas IL-27 and IFN-gamma decrease, splenocyte IL-17 production in WT mice. Eur J Immunol. (2010) 40:2549–56. 10.1002/eji.201040303 20623549 PMC3097107

[86] Liu H Loo KK Palaszynski K Ashouri J Lubahn DB Voskuhl RR. Estrogen receptor α mediates estrogen's immune protection in autoimmune disease. J Immunol. (2003) 171:6936–40. 10.4049/jimmunol.171.12.6936 14662901

[87] Lélu K Laffont S Delpy L Paulet P-E Périnat T Tschanz SA. Estrogen receptor α signaling in T lymphocytes is required for estradiol-mediated inhibition of Th1 and Th17 cell differentiation and protection against experimental autoimmune encephalomyelitis. J Immunol. (2011) 187:2386–93. 10.4049/jimmunol.1101578 21810607

[88] Chen R-Y Fan Y-M Zhang Q Liu S Li Q Ke G-L. Estradiol inhibits Th17 cell differentiation through inhibition of RORγT transcription by recruiting the ERα/REA complex to estrogen response elements of the RORγT promoter. J Immunol. (2015) 194:4019–28. 10.4049/jimmunol.1400806 25769926 PMC4390502

[89] Tyagi AM Srivastava K Mansoori MN Trivedi R Chattopadhyay N Singh D. Estrogen deficiency induces the differentiation of IL-17 secreting Th17 cells: a new candidate in the pathogenesis of osteoporosis. PLoS ONE (2012) 7:e44552. 10.1371/journal.pone.0044552 22970248 PMC3438183

[90] Molnár I Bohaty I Somogyiné-Vári É. High prevalence of increased interleukin-17A serum levels in postmenopausal estrogen deficiency. Menopause (2014) 21:749–52. 10.1097/GME.0000000000000125 24253487

[91] Gao Y Qian W-P Dark K Toraldo G Lin ASP Guldberg RE. Estrogen prevents bone loss through transforming growth factor β signaling in T cells. Proc Natl Acad Sci USA. (2004) 101:16618–23. 10.1073/pnas.0404888101 15531637 PMC534514

[92] Park H-J Park H-S Lee J-U Bothwell ALM Choi J-M. Sex-based selectivity of PPARγ regulation in Th1, Th2, and Th17 differentiation. Int J Mol Sci. (2016) 17:81347. 10.3390/ijms17081347 27548145 PMC5000743

[93] Aggelakopoulou M Kourepini E Paschalidis N Panoutsakopoulou V. ERβ in CD4^+^ T cells is crucial for ligand-mediated suppression of central nervous system autoimmunity. J Immunol. (2016) 196:4947–56. 10.4049/jimmunol.1600246 27183630

[94] Kitagawa Y Sakaguchi S. Molecular control of regulatory T cell development and function. Curr Opin Immunol. (2017) 49:64–70. 10.1016/j.coi.2017.10.002 29065384

[95] Li MO Rudensky AY. T cell receptor signalling in the control of regulatory T cell differentiation and function. Nat Rev Immunol. (2016) 16:220–33. 10.1038/nri.2016.26 27026074 PMC4968889

[96] Nie J Li YY Zheng SG Tsun A Li B. FOXP3^+^ Treg cells and gender bias in autoimmune diseases. Front Immunol. (2015) 6:493. 10.3389/fimmu.2015.00493 26441996 PMC4585344

[97] Polanczyk MJ Carson BD Subramanian S Afentoulis M Vandenbark AA Ziegler SF. Cutting edge: estrogen drives expansion of the CD4^+^CD25^+^ regulatory T cell compartment. J Immunol. (2004) 173:2227–30. 10.4049/jimmunol.173.4.2227 15294932

[98] Dinesh RK Hahn BH Singh RP. PD-1, gender, and autoimmunity. Autoimmun Rev. (2010) 9:583–7. 10.1016/j.autrev.2010.04.003 20433954 PMC2884990

[99] Prieto GA Rosenstein Y. Oestradiol potentiates the suppressive function of human CD4 CD25 regulatory T cells by promoting their proliferation. Immunology (2006) 118:58–65. 10.1111/j.1365-2567.2006.02339.x 16630023 PMC1782269

[100] Arruvito L Sanz M Banham AH Fainboim L. Expansion of CD4^+^CD25^+^ and FOXP3^+^ regulatory T cells during the follicular phase of the menstrual cycle: implications for human reproduction. J Immunol. (2007) 178:2572–8. 10.4049/jimmunol.178.4.2572 17277167

[101] Luo CY Wang L Sun C Li DJ. Estrogen enhances the functions of CD4^+^CD25^+^Foxp3^+^ regulatory T cells that suppress osteoclast differentiation and bone resorption *in vitro*. Cell Mol Immunol. (2011) 8:50–8. 10.1038/cmi.2010.54 21200384 PMC4002989

[102] Adurthi S Kumar MM Vinodkumar HS Mukherjee G Krishnamurthy H Acharya KK. Oestrogen Receptor-α binds the FOXP3 promoter and modulates regulatory T-cell function in human cervical cancer. Sci Rep. (2017) 7:17289. 10.1038/s41598-017-17102-w 29229929 PMC5725534

[103] Mo R Chen J Grolleau-Julius A Murphy HS Richardson BC Yung RL. Estrogen regulates CCR gene expression and function in T lymphocytes. J Immunol. (2005) 174:6023–9. 10.4049/jimmunol.174.10.6023 15879095

[104] Lengi AJ Phillips RA Karpuzoglu E Ahmed SA. Estrogen selectively regulates chemokines in murine splenocytes. J Leukoc Biol. (2007) 81:1065–74. 10.1189/jlb.0606391 17185357

[105] Andersson A Stubelius A Karlsson MN Engdahl C Erlandsson M Grahnemo L. Estrogen regulates T helper 17 phenotype and localization in experimental autoimmune arthritis. Arthritis Res Ther. (2015) 17:32. 10.1186/s13075-015-0548-y 25888974 PMC4355457

[106] Blanco P Ueno H Schmitt N. T follicular helper (Tfh) cells in lupus: activation and involvement in SLE pathogenesis. Eur J Immunol. (2016) 46:281–90. 10.1002/eji.201545760 26614103

[107] Park H-J Park H-S Lee J-U Bothwell ALM Choi J-M. Gender-specific differences in PPARγ regulation of follicular helper T cell responses with estrogen. Sci Rep. (2016) 6:28495. 10.1038/srep28495 27335315 PMC4917844

[108] Rider V Jones S Evans M Bassiri H Afsar Z Abdou NI. Estrogen increases CD40 ligand expression in T cells from women with systemic lupus erythematosus. J Rheumatol. (2001) 28:2644–9. 11764210

[109] Kincade PW Medina KL Payne KJ Rossi MI Tudor KS Yamashita Y. Early B-lymphocyte precursors and their regulation by sex steroids. Immunol Rev. (2000) 175:128–37. 10.1111/j.1600-065X.2000.imr017502.x 10933598

[110] Medina KL Garrett KP Thompson LF Rossi MI Payne KJ Kincade PW. Identification of very early lymphoid precursors in bone marrow and their regulation by estrogen. Nat Immunol. (2001) 2:718–24. 10.1038/90659 11477408

[111] Cohen-Solal JFG Jeganathan V Hill L Kawabata D Rodriguez-Pinto D Grimaldi C. Hormonal regulation of B-cell function and systemic lupus erythematosus. Lupus (2008) 17:528–32. 10.1177/0961203308089402 18539705

[112] Sthoeger ZM Chiorazzi N Lahita RG. Regulation of the immune response by sex hormones. I. *in vitro* effects of estradiol and testosterone on pokeweed mitogen-induced human B cell differentiation. J Immunol. (1988) 141:91–8. 3288699

[113] Kanda N Tamaki K. Estrogen enhances immunoglobulin production by human PBMCs. J Allergy Clin Immunol. (1999) 103:282–8. 9949320 10.1016/s0091-6749(99)70503-8

[114] Medina KL Smithson G Kincade PW. Suppression of B lymphopoiesis during normal pregnancy. J Exp Med. (1993) 178:1507–15. 8228804 10.1084/jem.178.5.1507 PMC2191236

[115] Masuzawa T Miyaura C Onoe Y Kusano K Ohta H Nozawa S. Estrogen deficiency stimulates B lymphopoiesis in mouse bone marrow. J Clin Invest. (1994) 94:1090–7. 10.1172/JCI117424 8083350 PMC295170

[116] Smithson G Medina K Ponting I Kincade PW. Estrogen suppresses stromal cell-dependent lymphopoiesis in culture. J Immunol. (1995) 155:3409–17. 7561035

[117] Smithson G Couse JF Lubahn DB Korach KS Kincade PW. The role of estrogen receptors and androgen receptors in sex steroid regulation of B lymphopoiesis. J Immunol. (1998) 161:27–34. 9647203

[118] Medina KL Strasser A Kincade PW. Estrogen influences the differentiation, proliferation, and survival of early B-lineage precursors. Blood (2000) 95:2059–67. 10706875

[119] Miyaura C Onoe Y Inada M Maki K Ikuta K Ito M. Increased B-lymphopoiesis by interleukin 7 induces bone loss in mice with intact ovarian function: similarity to estrogen deficiency. Proc Natl Acad Sci USA. (1997) 94:9360–5. 9256487 10.1073/pnas.94.17.9360 PMC23193

[120] Yokota T Oritani K Garrett KP Kouro T Nishida M Takahashi I. Soluble frizzled-related protein 1 is estrogen inducible in bone marrow stromal cells and suppresses the earliest events in lymphopoiesis. J Immunol. (2008) 181:6061–72. 10.4049/jimmunol.181.9.6061 18941195 PMC2735054

[121] Suurmond J Calise J Malkiel S Diamond B. DNA-reactive B cells in lupus. Curr Opin Immunol. (2016) 43:1–7. 10.1016/j.coi.2016.07.002 27504587 PMC5125853

[122] Malkiel S Barlev AN Atisha-Fregoso Y Suurmond J Diamond B. Plasma cell differentiation pathways in systemic lupus erythematosus. Front Immunol. (2018) 9:427. 10.3389/fimmu.2018.00427 29556239 PMC5845388

[123] Grimaldi CM Michael DJ Diamond B. Cutting edge: expansion and activation of a population of autoreactive marginal zone B cells in a model of estrogen-induced lupus. J Immunol. (2001) 167:1886–90. 10.4049/jimmunol.167.4.1886 11489967

[124] Grimaldi CM Cleary J Dagtas AS Moussai D Diamond B. Estrogen alters thresholds for B cell apoptosis and activation. J Clin Invest. (2002) 109:1625–33. 10.1172/JCI14873 12070310 PMC151010

[125] Grimaldi CM Jeganathan V Diamond B. Hormonal regulation of B cell development: 17 β-estradiol impairs negative selection of high-affinity DNA-reactive B cells at more than one developmental checkpoint. J Immunol. (2006) 176:2703–10. 10.4049/jimmunol.176.5.2703 16493025

[126] Bynoe MS Grimaldi CM Diamond B. Estrogen up-regulates Bcl-2 and blocks tolerance induction of naive B cells. Proc Natl Acad Sci USA. (2000) 97:2703–8. 10.1073/pnas.040577497 10694576 PMC15993

[127] Hill L Jeganathan V Chinnasamy P Grimaldi C Diamond B. Differential roles of estrogen receptors α and β in control of B-cell maturation and selection. Mol Med. (2011) 17:211–20. 10.2119/molmed.2010.00172 21107497 PMC3060981

[128] Jeganathan V Peeva E Diamond B. Hormonal milieu at time of B cell activation controls duration of autoantibody response. J Autoimmun. (2014) 53:46–54. 10.1016/j.jaut.2014.02.007 24685232 PMC4388304

[129] Rawlings DJ Metzler G Wray-Dutra M Jackson SW. Altered B cell signalling in autoimmunity. Nat Rev Immunol. (2017) 17:421–36. 10.1038/nri.2017.24 28393923 PMC5523822

[130] Panchanathan R Choubey D. Murine BAFF expression is up-regulated by estrogen and interferons: implications for sex bias in the development of autoimmunity. Mol Immunol. (2013) 53:15–23. 10.1016/j.molimm.2012.06.013 22784990 PMC3439561

[131] Bassi N Luisetto R Ghirardello A Gatto M Valente M Della Barbera M. 17-β-estradiol affects BLyS serum levels and the nephritogenic autoantibody network accelerating glomerulonephritis in NZB/WF1 mice. Lupus (2015) 24:382–91. 10.1177/0961203314559636 25801881

[132] Drehmer MN Suterio DG Muniz YCN de Souza IR Löfgren SE. BAFF Expression is Modulated by Female Hormones in Human Immune Cells. Biochem Genet. (2016) 54:722–30. 10.1007/s10528-016-9752-y 27306360

[133] Jones BG Penkert RR Xu B Fan Y Neale G Gearhart PJ. Binding of estrogen receptors to switch sites and regulatory elements in the immunoglobulin heavy chain locus of activated B cells suggests a direct influence of estrogen on antibody expression. Mol Immunol. (2016) 77:97–102. 10.1016/j.molimm.2016.07.015 27494228 PMC5010968

[134] Mohan C Putterman C. Genetics and pathogenesis of systemic lupus erythematosus and lupus nephritis. Nat Rev Nephrol. (2015) 11:329–41. 10.1038/nrneph.2015.33 25825084

[135] Perl A. Review: metabolic control of immune system activation in rheumatic diseases. Arthr Rheumatol. (2017) 69:2259–70. 10.1002/art.40223 28841779 PMC5711528

[136] Li J McMurray RW. Effects of estrogen receptor subtype-selective agonists on autoimmune disease in lupus-prone NZB/NZW F1 mouse model. Clin Immunol. (2007) 123:219–26. 10.1016/j.clim.2007.01.008 17336162

[137] Bynoté KK Hackenberg JM Korach KS Lubahn DB Lane PH Gould KA. Estrogen receptor-α deficiency attenuates autoimmune disease in (NZB x NZW)F1 mice. Genes Immun. (2008) 9:137–52. 10.1038/sj.gene.6364458 18200028

[138] Svenson JL EuDaly J Ruiz P Korach KS Gilkeson GS. Impact of estrogen receptor deficiency on disease expression in the NZM2410 lupus prone mouse. Clin Immunol. (2008) 128:259–68. 10.1016/j.clim.2008.03.508 18514033 PMC4778964

[139] Feng F Nyland J Banyai M Tatum A Silverstone AE Gavalchin J. The induction of the lupus phenotype by estrogen is via an estrogen receptor-α-dependent pathway. Clin Immunol. (2010) 134:226–36. 10.1016/j.clim.2009.10.004 19926524

[140] Chen Y Cuda C Morel L. Genetic determination of T cell help in loss of tolerance to nuclear antigens. J Immunol. (2005) 174:7692–702. 10.4049/jimmunol.174.12.7692 15944270

[141] Perry DJ Yin Y Telarico T Baker HV Dozmorov I Perl A. Murine lupus susceptibility locus Sle1c2 mediates CD4^+^ T cell activation and maps to estrogen-related receptor γ. J Immunol. (2012) 189:793–803. 10.4049/jimmunol.1200411 22711888 PMC3392454

[142] Yoachim SD Nuxoll JS Bynoté KK Gould KA. Estrogen receptor α signaling promotes Sle1-induced loss of tolerance and immune cell activation and is responsible for sex bias in B6.Sle1 congenic mice. Clin Immunol. (2015) 158:153–66. 10.1016/j.clim.2015.03.026 25862391 PMC4465054

[143] Tabor DE Gould KA Estrogen receptor α promotes lupus in (NZB × NZW)F1 mice in a B cell intrinsic manner. Clin Immunol. (2017) 174:41–52. 10.1016/j.clim.2016.10.011 27989899 PMC5316311

[144] Moulton VR Tsokos GC. T cell signaling abnormalities contribute to aberrant immune cell function and autoimmunity. J Clin Invest. (2015) 125:2220–7. 10.1172/JCI78087 25961450 PMC4497749

[145] Cutolo M Sulli A Straub RH. Estrogen metabolism and autoimmunity. Autoimmun Rev. (2012) 11:A460–4. 10.1016/j.autrev.2011.11.014 22155198

[146] Kassi EN Vlachoyiannopoulos PG Moutsopoulos HM Sekeris CE Moutsatsou P. Molecular analysis of estrogen receptor α and β in lupus patients. Eur J Clin Invest. (2001) 31:86–93. 10.1046/j.1365-2362.2001.00762.x 11168443

[147] Inui A Ogasawara H Naito T Sekigawa I Takasaki Y Hayashida Y. Estrogen receptor expression by peripheral blood mononuclear cells of patients with systemic lupus erythematosus. Clin Rheumatol. (2007) 26:1675–8. 10.1007/s10067-007-0568-3 17874259

[148] Lee YJ Shin KS Kang SW Lee CK Yoo B Cha HS. Association of the oestrogen receptor α gene polymorphisms with disease onset in systemic lupus erythematosus. Ann Rheum Dis. (2004) 63:1244–9. 10.1136/ard.2003.012583 15361380 PMC1754755

[149] Kassi E Vlachoyiannopoulos PG Kominakis A Kiaris H Moutsopoulos HM Moutsatsou P. Estrogen receptor α gene polymorphism and systemic lupus erythematosus: a possible risk? Lupus (2005) 14:391–8. 10.1191/0961203305lu2104oa 15934440

[150] Johansson M Arlestig L Möller B Smedby T Rantapää-Dahlqvist S. Oestrogen receptor α gene polymorphisms in systemic lupus erythematosus. Ann Rheum Dis. (2005) 64:1611–7. 10.1136/ard.2004.032425 15817658 PMC1755265

[151] Kisiel BM Kosinska J Wierzbowska M Rutkowska-Sak L Musiej-Nowakowska E Wudarski M. Differential association of juvenile and adult systemic lupus erythematosus with genetic variants of oestrogen receptors α and β. Lupus (2011) 20:85–9. 10.1177/0961203310381514 20961965

[152] Drehmer MN Andrade D Pereira IA Marrero AR Muniz YCN de Souza IR. Estrogen receptor α gene (ESR1) polymorphism can contribute to clinical findings in systemic lupus erythematosus patients. Lupus (2017) 26:294–8. 10.1177/0961203316668041 27681518

[153] Teruel M Sawalha AH. Epigenetic variability in systemic lupus erythematosus: what we learned from genome-wide DNA methylation studies. Curr Rheumatol Rep. (2017) 19:32. 10.1007/s11926-017-0657-5 28470479 PMC5819620

[154] Weeding E Sawalha AH. Deoxyribonucleic acid methylation in systemic lupus erythematosus: implications for future clinical practice. Front Immunol. (2018) 9:875. 10.3389/fimmu.2018.00875 29740453 PMC5928134

[155] Gorjestani S Rider V Kimler BF Greenwell C Abdou NI. Extracellular signal-regulated kinase 1/2 signalling in SLE T cells is influenced by oestrogen and disease activity. Lupus (2008) 17:548–54. 10.1177/0961203307087982 18539708

[156] Pan W Zhu S Yuan M Cui H Wang L Luo X. MicroRNA-21 and microRNA-148a contribute to DNA hypomethylation in lupus CD4^+^ T cells by directly and indirectly targeting DNA methyltransferase 1. J Immunol. (2010) 184:6773–81. 10.4049/jimmunol.0904060 20483747

[157] Strickland FM Hewagama A Lu Q Wu A Hinderer R Webb R. Environmental exposure, estrogen and two X chromosomes are required for disease development in an epigenetic model of lupus. J Autoimmun. (2012) 38:J135–43. 10.1016/j.jaut.2011.11.001 22142890 PMC3312994

[158] Rider V Jones SR Evans M Abdou NI. Molecular mechanisms involved in the estrogen-dependent regulation of calcineurin in systemic lupus erythematosus T cells. Clin Immunol. (2000) 95:124–34. 10.1006/clim.2000.4844 10779406

[159] Rider V Li X Peterson G Dawson J Kimler BF Abdou NI. Differential expression of estrogen receptors in women with systemic lupus erythematosus. J Rheumatol. (2006) 33:1093–101. 16755656

[160] Ward JM Rider V Abdou NI Kimler B. Estradiol differentially regulates calreticulin: a potential link with abnormal T cell function in systemic lupus erythematosus? Lupus (2013) 22:583–96. 10.1177/0961203313482742 23535532 PMC4072130

[161] Young NA Friedman AK Kaffenberger B Rajaram MVS Birmingham DJ Rovin BH. Novel estrogen target gene ZAS3 is overexpressed in systemic lupus erythematosus. Mol Immunol. (2013) 54:23–31. 10.1016/j.molimm.2012.10.026 23178823 PMC3913539

[162] Kim W-U Min S-Y Hwang S-H Yoo S-A Kim K-J Cho C-S. Effect of oestrogen on T cell apoptosis in patients with systemic lupus erythematosus. Clin Exp Immunol. (2010) 161:453–8. 10.1111/j.1365-2249.2010.04194.x 20529085 PMC2962962

[163] Rastin M Hatef MR Tabasi N Mahmoudi M. The pathway of estradiol-induced apoptosis in patients with systemic lupus erythematosus. Clin Rheumatol. (2012) 31:417–24. 10.1007/s10067-011-1821-3 21837431

[164] Colasanti T Maselli A Conti F Sanchez M Alessandri C Barbati C. Autoantibodies to estrogen receptor α interfere with T lymphocyte homeostasis and are associated with disease activity in systemic lupus erythematosus. Arthritis Rheum. (2012) 64:778–87. 10.1002/art.33400 21968947

[165] Walters E Rider V Abdou NI Greenwell C Svojanovsky S Smith P. Estradiol targets T cell signaling pathways in human systemic lupus. Clin Immunol. (2009) 133:428–36. 10.1016/j.clim.2009.09.002 19793680 PMC2783703

[166] Rider V Abdou NI Kimler BF Lu N Brown S Fridley BL. Gender bias in human systemic lupus erythematosus: a problem of steroid receptor action? Front Immunol. (2018) 9:611. 10.3389/fimmu.2018.00611 29643853 PMC5882779

[167] Giang S La Cava A. Regulatory T cells in SLE: biology and use in treatment. Curr Rheumatol Rep. (2016) 18:67. 10.1007/s11926-016-0616-6 27704250

[168] Mizui M Tsokos GC. Targeting regulatory T cells to treat patients with systemic lupus erythematosus. Front Immunol (2018) 9:786. 10.3389/fimmu.2018.00786 29755456 PMC5932391

[169] Kimura A Kishimoto T. IL-6: regulator of Treg/Th17 balance. Eur J Immunol. (2010) 40:1830–5. 10.1002/eji.201040391 20583029

[170] Yang XO Nurieva R Martinez GJ Kang HS Chung Y Pappu BP. Molecular antagonism and plasticity of regulatory and inflammatory T cell programs. Immunity (2008) 29:44–56. 10.1016/j.immuni.2008.05.007 18585065 PMC2630532

[171] Linker-Israeli M Deans RJ Wallace DJ Prehn J Ozeri-Chen T Klinenberg JR. Elevated levels of endogenous IL-6 in systemic lupus erythematosus. a putative role in pathogenesis. J Immunol. (1991) 147:117–23. 2051017

[172] Ripley BJ Goncalves B Isenberg DA Latchman DS Rahman A. Raised levels of interleukin 6 in systemic lupus erythematosus correlate with anaemia. Ann Rheum Dis. (2005) 64:849–53. 10.1136/ard.2004.022681 15897306 PMC1755543

[173] Sabry A El-Agroudy A Sheashaa H Hawas S El-Shahat FB Barakat N. Co-administration of ketoconazole and tacrolimus therapy: a transplanted rat model. Int Urol Nephrol. (2006) 38:713–8. 10.1007/s11255-006-0062-x 17160448

[174] Li Y Tucci M Narain S Barnes EV Sobel ES Segal MS. Urinary biomarkers in lupus nephritis. Autoimmun Rev. (2006) 5:383–8. 10.1016/j.autrev.2005.10.006 16890891

[175] Isse K Specht SM Lunz JG Kang L-I Mizuguchi Y Demetris AJ. Estrogen stimulates female biliary epithelial cell interleukin-6 expression in mice and humans. Hepatology (2010) 51:869–80. 10.1002/hep.23386 20043322

[176] Olivieri F Bonafè M Cavallone L Giovagnetti S Marchegiani F Cardelli M. The−174 C/G locus affects *in vitro/in vivo* IL-6 production during aging. Exp Gerontol. (2002) 37:309–14. 10.1016/S0531-5565(01)00197-8 11772517

[177] Mao X Wu Y Diao H Hao J Tian G Jia Z. Interleukin-6 promotes systemic lupus erythematosus progression with Treg suppression approach in a murine systemic lupus erythematosus model. Clin Rheumatol. (2014) 33:1585–93. 10.1007/s10067-014-2717-9 24928344

[178] Lu R Munroe ME Guthridge JM Bean KM Fife DA Chen H. Dysregulation of innate and adaptive serum mediators precedes systemic lupus erythematosus classification and improves prognostic accuracy of autoantibodies. J Autoimmun. (2016) 74:182–93. 10.1016/j.jaut.2016.06.001 27338520 PMC5079766

[179] Munroe ME Lu R Zhao YD Fife DA Robertson JM Guthridge JM. Altered type II interferon precedes autoantibody accrual and elevated type I interferon activity prior to systemic lupus erythematosus classification. Ann Rheum Dis. (2016) 75:2014–21. 10.1136/annrheumdis-2015-208140 27088255 PMC4959992

[180] Panchanathan R Shen H Zhang X Ho S-M Choubey D. Mutually positive regulatory feedback loop between interferons and estrogen receptor-α in mice: implications for sex bias in autoimmunity. PLoS ONE (2010) 5:e10868. 10.1371/journal.pone.0010868 20526365 PMC2878324

[181] Michaelson JS Wisniacki N Burkly LC Putterman C. Role of TWEAK in lupus nephritis: a bench-to-bedside review. J Autoimmun. (2012) 39:130–42. 10.1016/j.jaut.2012.05.003 22727560 PMC3428508

[182] Xue L Liu Z Hu J Huang J Wen J Liu Z. Estrogen-induced expression of tumor necrosis factor-like weak inducer of apoptosis through ERα accelerates the progression of lupus nephritis. Rheumatology (2016) 55:1880–8. 10.1093/rheumatology/kew248 27354685

[183] Corradetti C Jog NR Cesaroni M Madaio M Caricchio R. Estrogen receptor α signaling exacerbates immune-mediated nephropathies through alteration of metabolic activity. J Immunol. (2018) 200:512–22. 10.4049/jimmunol.1700770 29237779 PMC5760359

[184] Scott JL Wirth JR Eudaly J Ruiz P Cunningham MA Complete knockout of estrogen receptor α is not directly protective in murine lupus. Clin Immunol. (2017) 183:132–41. 10.1016/j.clim.2017.08.010 28822833 PMC6261466

[185] Khan D Ansar Ahmed S. The immune system is a natural target for estrogen action: opposing effects of estrogen in two prototypical autoimmune diseases. Front Immunol. (2016) 6:635. 10.3389/fimmu.2015.00635 26779182 PMC4701921

[186] Confavreux C Hutchinson M Hours MM Cortinovis-Tourniaire P Moreau T. Rate of pregnancy-related relapse in multiple sclerosis. pregnancy in multiple sclerosis group. N Engl J Med. (1998) 339:285–91. 10.1056/NEJM199807303390501 9682040

[187] Sicotte NL Liva SM Klutch R Pfeiffer P Bouvier S Odesa S. Treatment of multiple sclerosis with the pregnancy hormone estriol. Ann Neurol. (2002) 52:421–8. 10.1002/ana.10301 12325070

[188] Bebo BF Fyfe-Johnson A Adlard K Beam AG Vandenbark AA Offner H. Low-dose estrogen therapy ameliorates experimental autoimmune encephalomyelitis in two different inbred mouse strains. J Immunol. (2001) 166:2080–9. 10.4049/jimmunol.166.3.2080 11160259

[189] Duncan GS Brenner D Tusche MW Brüstle A Knobbe CB Elia AJ. 2-Methoxyestradiol inhibits experimental autoimmune encephalomyelitis through suppression of immune cell activation. Proc Natl Acad Sci USA. (2012) 109:21034–9. 10.1073/pnas.1215558110 23213242 PMC3529073

[190] Pozzilli C Falaschi P Mainero C Martocchia A D'Urso R Proietti A. MRI in multiple sclerosis during the menstrual cycle: relationship with sex hormone patterns. Neurology (1999) 53:622–4. 10449131 10.1212/wnl.53.3.622

[191] Spence RD Voskuhl RR. Neuroprotective effects of estrogens and androgens in CNS inflammation and neurodegeneration. Front Neuroendocrinol. (2012) 33:105–15. 10.1016/j.yfrne.2011.12.001 22209870 PMC3616506

[192] Itoh N Kim R Peng M DiFilippo E Johnsonbaugh H MacKenzie-Graham A. Bedside to bench to bedside research: estrogen receptor β ligand as a candidate neuroprotective treatment for multiple sclerosis. J Neuroimmunol. (2017) 304:63–71. 10.1016/j.jneuroim.2016.09.017 27771018 PMC5806698

[193] Voskuhl RR Wang HJ Wu TCJ Sicotte NL Nakamura K Kurth F. Estriol combined with glatiramer acetate for women with relapsing-remitting multiple sclerosis: a randomised, placebo-controlled, phase 2 trial. Lancet Neurol. (2016) 15:35–46. 10.1016/S1474-4422(15)00322-1 26621682

[194] Seifert HA Benedek G Nguyen H Kent G Vandenbark AA Offner H. Estrogen protects both sexes against EAE by promoting common regulatory cell subtypes independent of endogenous estrogen. Metab Brain Dis. (2017) 32:1747–54. 10.1007/s11011-017-0063-8 28689297 PMC5650507

[195] Wang C Dehghani B Li Y Kaler LJ Vandenbark AA Offner H. Oestrogen modulates experimental autoimmune encephalomyelitis and interleukin-17 production via programmed death 1. Immunology (2009) 126:329–35. 10.1111/j.1365-2567.2008.03051.x 19302141 PMC2669813

[196] Polanczyk MJ Hopke C Vandenbark AA Offner H. Treg suppressive activity involves estrogen-dependent expression of programmed death-1 (PD-1). Int Immunol. (2007) 19:337–43. 10.1093/intimm/dxl151 17267414

[197] Haghmorad D Amini AA Mahmoudi MB Rastin M Hosseini M Mahmoudi M. Pregnancy level of estrogen attenuates experimental autoimmune encephalomyelitis in both ovariectomized and pregnant C57BL/6 mice through expansion of Treg and Th2 cells. J Neuroimmunol. (2014) 277:85–95. 10.1016/j.jneuroim.2014.10.004 25457839

[198] Alpízar-Rodríguez D Pluchino N Canny G Gabay C Finckh A. The role of female hormonal factors in the development of rheumatoid arthritis. Rheumatology (2017) 56:1254–63. 10.1093/rheumatology/kew318 27686101

[199] Islander U Jochems C Lagerquist MK Forsblad-d'Elia H Carlsten H. Estrogens in rheumatoid arthritis; the immune system and bone. Mol Cell Endocrinol. (2011) 335:14–29. 10.1016/j.mce.2010.05.018 20685609

[200] Alpízar-Rodríguez D Finckh A. Environmental factors and hormones in the development of rheumatoid arthritis. Semin Immunopathol. (2017) 39:461–8. 10.1007/s00281-017-0624-2 28451785

[201] Straub RH. The complex role of estrogens in inflammation. Endocr Rev. (2007) 28:521–74. 10.1210/er.2007-0001 17640948

[202] Furie R. Dehydroepiandrosterone and biologics in the treatment of systemic lupus erythematosus. Curr Rheumatol Rep. (2000) 2:44–50. 10.1007/s11926-996-0068-5 11123039

[203] Karpuzoglu E Zouali M. The multi-faceted influences of estrogen on lymphocytes: toward novel immuno-interventions strategies for autoimmunity management. Clin Rev Allergy Immunol. (2011) 40:16–26. 10.1007/s12016-009-8188-0 19943123

[204] Nilsson S Koehler KF Gustafsson J-Å. Development of subtype-selective oestrogen receptor-based therapeutics. Nat Rev Drug Discov. (2011) 10:778–92. 10.1038/nrd3551 21921919

[205] An K-C. Selective estrogen receptor modulators. Asian Spine J. (2016) 10:787–91. 10.4184/asj.2016.10.4.787 27559463 PMC4995266

[206] Apelgren LD Bailey DL Fouts RL Short L Bryan N Evans GF. The effect of a selective estrogen receptor modulator on the progression of spontaneous autoimmune disease in MRL lpr/lpr mice. Cell Immunol. (1996) 173:55–63. 10.1006/cimm.1996.0251 8871601

[207] Wu WM Suen JL Lin BF Chiang BL. Tamoxifen alleviates disease severity and decreases double negative T cells in autoimmune MRL-lpr/lpr mice. Immunology (2000) 100:110–8. 10809966 10.1046/j.1365-2567.2000.00998.x PMC2326982

[208] Erlandsson MC Gömöri E Taube M Carlsten H. Effects of raloxifene, a selective estrogen receptor modulator, on thymus, T cell reactivity, and inflammation in mice. Cell Immunol. (2000) 205:103–9. 10.1006/cimm.2000.1719 11104582

[209] Bernardi AI Andersson A Grahnemo L Nurkkala-Karlsson M Ohlsson C Carlsten H. Effects of lasofoxifene and bazedoxifene on B cell development and function. Immun Inflamm Dis. (2014) 2:214–25. 10.1002/iid3.37 25866629 PMC4386916

[210] Wu X Tong B Yang Y Luo J Yuan X Wei Z. Arctigenin functions as a selective agonist of estrogen receptor β to restrict mTORC1 activation and consequent Th17 differentiation. Oncotarget (2016) 7:83893–906. 10.18632/oncotarget.13338 27863380 PMC5356633

[211] Polari L Wiklund A Sousa S Kangas L Linnanen T Härkönen P. SERMs promote anti-inflammatory signaling and phenotype of CD14^+^ cells. Inflammation (2018) 41, 1157–71. 10.1007/s10753-018-0763-1 29574654 PMC6061028

[212] Petri MA Mease PJ Merrill JT Lahita RG Iannini MJ Yocum DE. Effects of prasterone on disease activity and symptoms in women with active systemic lupus erythematosus. Arthritis Rheum. (2004) 50:2858–68. 10.1002/art.20427 15452837

[213] Abdou NI Rider V Greenwell C Li X Kimler BF. Fulvestrant (Faslodex), an estrogen selective receptor downregulator, in therapy of women with systemic lupus erythematosus. clinical, serologic, bone density, and T cell activation marker studies: a double-blind placebo-controlled trial. J Rheumatol. (2008) 35:797. 18381791

[214] Hughes GC. Progesterone and autoimmune disease. Autoimmun Rev. (2012) 11:A502–14. 10.1016/j.autrev.2011.12.003 22193289 PMC3431799

[215] Greenstein B Roa R Daher Y Nunn E Greenstein A Khamashta M. Estrogen and progesterone receptors in murine models of systemic lupus erythematosus. Int Immunopharmacol. (2001) 1:1025–35. 10.1016/S1567-5769(01)00034-0 11407299

[216] Tan IJ Peeva E Zandman-Goddard G. Hormonal modulation of the immune system—a spotlight on the role of progestogens. Autoimmun Rev. (2015) 14:536–42. 10.1016/j.autrev.2015.02.004 25697984

[217] Gubbels Bupp MR Jorgensen TN. Androgen-induced immunosuppression. Front Immunol. (2018) 9:794. 10.3389/fimmu.2018.00794 29755457 PMC5932344

[218] Pakpoor J Goldacre R Goldacre MJ. Associations between clinically diagnosed testicular hypofunction and systemic lupus erythematosus: a record linkage study. Clin Rheumatol. (2018) 37:559–62. 10.1007/s10067-017-3873-5 29101673 PMC5775979

[219] Procaccini C Pucino V Mantzoros CS Matarese G. Leptin in autoimmune diseases. Metab Clin Exp. (2015) 64:92–104. 10.1016/j.metabol.2014.10.014 25467840

[220] Naylor C Petri WA. Leptin regulation of immune responses. Trends Mol Med. (2016) 22:88–98. 10.1016/j.molmed.2015.12.001 26776093

[221] La Cava A. Leptin in inflammation and autoimmunity. Cytokine (2017) 98:51–8. 10.1016/j.cyto.2016.10.011 27916613 PMC5453851

[222] Pérez-Pérez A Vilariño-García T Fernández-Riejos P Martín-González J Segura-Egea JJ Sánchez-Margalet V. Role of leptin as a link between metabolism and the immune system. Cytokine Growth Factor Rev. (2017) 35:71–84. 10.1016/j.cytogfr.2017.03.001 28285098

[223] Francisco V Pino J Campos-Cabaleiro V Ruiz-Fernández C Mera A Gonzalez-Gay MA. Obesity, fat mass and immune system: role for leptin. Front Physiol. (2018) 9:640. 10.3389/fphys.2018.00640 29910742 PMC5992476

[224] Lord GM Matarese G Howard JK Baker RJ Bloom SR Lechler RI. Leptin modulates the T-cell immune response and reverses starvation-induced immunosuppression. Nature (1998) 394:897–901. 10.1038/29795 9732873

[225] Yu Y Liu Y Shi F-D Zou H Matarese G La Cava A. Cutting edge: leptin-induced RORγt expression in CD4^+^ T cells promotes Th17 responses in systemic lupus erythematosus. J Immunol. (2013) 190:3054–8. 10.4049/jimmunol.1203275 23447682 PMC3608794

[226] Procaccini C De Rosa V Galgani M Carbone F Cassano S Greco D. Leptin-induced mTOR activation defines a specific molecular and transcriptional signature controlling CD4^+^ effector T cell responses. J Immunol. (2012) 189:2941–53. 10.4049/jimmunol.1200935 22904304

[227] Gerriets VA Danzaki K Kishton RJ Eisner W Nichols AG Saucillo DC. Leptin directly promotes T-cell glycolytic metabolism to drive effector T-cell differentiation in a mouse model of autoimmunity. Eur J Immunol. (2016) 46:1970–83. 10.1002/eji.201545861 27222115 PMC5154618

[228] Lourenço EV Liu A Matarese G La Cava A. Leptin promotes systemic lupus erythematosus by increasing autoantibody production and inhibiting immune regulation. Proc Natl Acad Sci USA. (2016) 113:10637–42. 10.1073/pnas.1607101113 27588900 PMC5035847

[229] Amarilyo G Iikuni N Liu A Matarese G La Cava A. Leptin enhances availability of apoptotic cell-derived self-antigen in systemic lupus erythematosus. PLoS ONE (2014) 9:e112826. 10.1371/journal.pone.0112826 25401752 PMC4234630

[230] De Rosa V Procaccini C Calì G Pirozzi G Fontana S Zappacosta S. A key role of leptin in the control of regulatory T cell proliferation. Immunity (2007) 26:241–55. 10.1016/j.immuni.2007.01.011 17307705

[231] Lam QLK Wang S Ko OKH Kincade PW Lu L. Leptin signaling maintains B-cell homeostasis via induction of Bcl-2 and Cyclin D1. Proc Natl Acad Sci USA. (2010) 107:13812–7. 10.1073/pnas.1004185107 20643953 PMC2922219

[232] Agrawal S Gollapudi S Su H Gupta S. Leptin activates human B cells to secrete TNF-α, IL-6, and IL-10 via JAK2/STAT3 and p38MAPK/ERK1/2 signaling pathway. J Clin Immunol. (2011) 31:472–8. 10.1007/s10875-010-9507-1 21243519 PMC3132280

[233] Lateef A Petri M. Hormone replacement and contraceptive therapy in autoimmune diseases. J Autoimmun. (2012) 38:J170–6. 10.1016/j.jaut.2011.11.002 22261500

[234] Williams WV. Hormonal contraception and the development of autoimmunity: a review of the literature. Linacre Q. (2017) 84:275–95. 10.1080/00243639.2017.1360065 28912620 PMC5592309

[235] Buyon JP Petri MA Kim MY Kalunian KC Grossman J Hahn BH. The effect of combined estrogen and progesterone hormone replacement therapy on disease activity in systemic lupus erythematosus: a randomized trial. Ann Intern Med. (2005) 142:953–62. 10.7326/0003-4819-142-12_Part_1-200506210-00004 15968009

[236] Petri M Kim MY Kalunian KC Grossman J Hahn BH Sammaritano LR. Combined oral contraceptives in women with systemic lupus erythematosus. N Engl J Med. (2005) 353:2550–8. 10.1056/NEJMoa051135 16354891

[237] Vieira CS Pereira FV de Sá MFS Paulo LJ Martins WP Ferriani RA. Tibolone in postmenopausal women with systemic lupus erythematosus: a pilot study. Maturitas (2009) 62:311–6. 10.1016/j.maturitas.2008.12.021 19193505

[238] Panay N Hamoda H Arya R Savvas M. The 2013 British Menopause Society & Women's Health Concern recommendations on hormone replacement therapy, The 2013 British Menopause Society & Women's Health Concern recommendations on hormone replacement therapy. Menopause Int. (2013) 19:59–68. 10.1177/1754045313489645 23761319

[239] Lindsay R Gallagher JC Kagan R Pickar JH Constantine G. Efficacy of tissue-selective estrogen complex of bazedoxifene/conjugated estrogens for osteoporosis prevention in at-risk postmenopausal women. Fertil Steril. (2009) 92:1045–52. 10.1016/j.fertnstert.2009.02.093 19635616

[240] Lobo RA Pinkerton JV Gass MLS Dorin MH Ronkin S Pickar JH. Evaluation of bazedoxifene/conjugated estrogens for the treatment of menopausal symptoms and effects on metabolic parameters and overall safety profile. Fertil Steril. (2009) 92:1025–38. 10.1016/j.fertnstert.2009.03.113 19635615

[241] Pickar JH Komm BS. Selective estrogen receptor modulators and the combination therapy conjugated estrogens/bazedoxifene: a review of effects on the breast. Post Reprod Health (2015) 21:112–21. 10.1177/2053369115599090 26289836

[242] Andersson A Bernardi AI Nurkkala-Karlsson M Stubelius A Grahnemo L Ohlsson C. Suppression of experimental arthritis and associated bone loss by a tissue-selective estrogen complex. Endocrinology (2016) 157:1013–20. 10.1210/en.2015-1820 26745543

[243] Zhang Y Saha S Rosenfeld G Gonzalez J Pepeljugoski KP Peeva E. Raloxifene modulates estrogen-mediated B cell autoreactivity in NZB/W F1 mice. J Rheumatol. (2010) 37:1646–57. 10.3899/jrheum.090911 20551107

[244] Nordqvist J Bernardi A Islander U Carlsten H. Effects of a tissue-selective estrogen complex on B lymphopoiesis and B cell function. Immunobiology (2017) 222:918–23. 10.1016/j.imbio.2017.05.013 28551078

[245] Wu G Xu R Zhang P Xiao T Fu Y Zhang Y. Estrogen regulates stemness and senescence of bone marrow stromal cells to prevent osteoporosis via ERβ-SATB2 pathway. J Cell Physiol. (2017) 233:4194–204. 10.1002/jcp.26233 29030963

[246] Borba VV Zandman-Goddard G Shoenfeld Y. Prolactin and autoimmunity. Front Immunol. (2018) 9:73. 10.3389/fimmu.2018.00073 29483903 PMC5816039

[247] McMahon M Skaggs BJ Sahakian L Grossman J FitzGerald J Ragavendra N. High plasma leptin levels confer increased risk of atherosclerosis in women with systemic lupus erythematosus, and are associated with inflammatory oxidised lipids. Ann Rheum Dis. (2011) 70:1619–24. 10.1136/ard.2010.142737 21670088 PMC3147230

